# Conserved Genetic Interactions between Ciliopathy Complexes Cooperatively Support Ciliogenesis and Ciliary Signaling

**DOI:** 10.1371/journal.pgen.1005627

**Published:** 2015-11-05

**Authors:** Laura E. Yee, Francesc R. Garcia-Gonzalo, Rachel V. Bowie, Chunmei Li, Julie K. Kennedy, Kaveh Ashrafi, Oliver E. Blacque, Michel R. Leroux, Jeremy F. Reiter

**Affiliations:** 1 Department of Biochemistry and Biophysics, University of California, San Francisco, San Francisco, California, United States of America; 2 School of Biomolecular and Biomedical Science, UCD Conway Institute, University College Dublin, Belfield, Dublin, Ireland; 3 Department of Molecular Biology and Biochemistry and Centre for Cell Biology, Development, and Disease, Simon Fraser University, Burnaby, British Columbia, Canada; 4 Department of Physiology, University of California, San Francisco, San Francisco, California, United States of America; Stanford University School of Medicine, UNITED STATES

## Abstract

Mutations in genes encoding cilia proteins cause human ciliopathies, diverse disorders affecting many tissues. Individual genes can be linked to ciliopathies with dramatically different phenotypes, suggesting that genetic modifiers may participate in their pathogenesis. The ciliary transition zone contains two protein complexes affected in the ciliopathies Meckel syndrome (MKS) and nephronophthisis (NPHP). The BBSome is a third protein complex, affected in the ciliopathy Bardet-Biedl syndrome (BBS). We tested whether mutations in MKS, NPHP and BBS complex genes modify the phenotypic consequences of one another in both *C*. *elegans* and mice. To this end, we identified TCTN-1, the *C*. *elegans* ortholog of vertebrate MKS complex components called Tectonics, as an evolutionarily conserved transition zone protein. Neither disruption of TCTN-1 alone or together with MKS complex components abrogated ciliary structure in *C*. *elegans*. In contrast, disruption of TCTN-1 together with either of two NPHP complex components, NPHP-1 or NPHP-4, compromised ciliary structure. Similarly, disruption of an NPHP complex component and the BBS complex component BBS-5 individually did not compromise ciliary structure, but together did. As in nematodes, disrupting two components of the mouse MKS complex did not cause additive phenotypes compared to single mutants. However, disrupting both Tctn1 and either Nphp1 or Nphp4 exacerbated defects in ciliogenesis and cilia-associated developmental signaling, as did disrupting both Tctn1 and the BBSome component Bbs1. Thus, we demonstrate that ciliary complexes act in parallel to support ciliary function and suggest that human ciliopathy phenotypes are altered by genetic interactions between different ciliary biochemical complexes.

## Author Summary

Ciliopathies, diseases arising from defects in the functions of primary cilia, have many different manifestations and vary dramatically in severity. How genetics influence ciliopathy phenotypes is poorly understood. Building off of our increasing knowledge of how different biochemical complexes contribute to ciliary function, we investigated how ciliopathy-associated genes interact to support ciliogenesis. Using a combination of nematode and mouse genetics, we found that genes encoding components of different biochemical complexes interact, whereas genes encoding different components within a single complex do not. These results revealed overlapping ciliary functions of biochemically distinct proteins complexes such as the BBSome, the transition zone MKS complex and the transition zone NPHP complex. This work indicates the genetic interactions that may alter the phenotypic consequences of human ciliopathy mutations.

## Introduction

Despite accelerating success in identifying genetic variations, the relationship between genotype and phenotype in humans often remains obscure. Even for many Mendelian diseases, the expressivity of disease alleles is not always predictable, indicating that additional genetic and epigenetic influences modify the disease-causing mutations. The genetic influences could include many common genetic variants with small effects, rare variants with large effects, and allele-specific gene-gene interactions. These genetic interactions can modify associated phenotypes through changes in gene expression, protein interactions, and effects on overlapping functions [1–4].

Ciliopathies, which arise from disrupted ciliary function, are prominent examples of disorders with complex inheritance. The clinical manifestations of ciliopathies are often variable and overlapping. For example, Meckel syndrome (MKS [MIM 249000]) is characterized by cystic kidney dysplasia, polydactyly, and occipital meningoencephalocoele. Nephronophthisis (NPHP [MIM 256100]), the most common genetic cause of renal failure in children, is characterized by cystic kidney dysplasia without limb or brain malformations. Bardet-Biedl syndrome **(**BBS [MIM 209900]), a disorder associated with at least nineteen loci, is characterized by cystic kidney dysplasia, polydactyly, retinal degeneration, obesity, and learning difficulties. Although BBS has been traditionally considered an autosomal recessive disorder affecting a single locus, in some instances three mutations in two loci are required to cause disease [5–9]. Thus, mutations in two loci can be required for ciliopathy penetrance.

As reflected by the broad spectrum of ciliopathy-associated phenotypes, primary cilia play numerous, often tissue-specific, roles in mammals. For example, cilia sense odorants and light, control growth in the kidney, and transduce the Hedgehog (Hh) signaling pathway, thereby playing integral functions in embryonic development and adult tissue homeostasis [10]. To transduce signals, cilia maintain compositions distinct from those of other cellular compartments. How cilia exclude some proteins while allowing others access remains a fundamental question [11]. Previous work has suggested that the transition zone, a region at the base of the cilium between the basal body and the axoneme proper, may be involved in this process [12–16]. The transition zone is defined ultrastructurally by the presence of Y-links that connect the microtubule core to the ciliary membrane [17, 18]. In mice, the proteins Tctn1, Tctn2, Tmem231, Tmem67, Mks1, B9d1, B9d2, Cep290 and Cc2d2a co-localize to the transition zone and form a large biochemical complex, the MKS complex [12, 16, 19]. A biochemically distinct complex that includes Nphp1 and Nphp4, termed the NPHP complex, also localizes to the transition zone [20, 21].

Mammals possess cilia on many cell types, whereas cilia in *Caenorhabditis elegans* are confined to the dendritic tips of 60 select sensory neurons in hermaphrodites. As in mammals, the *C*. *elegans* orthologs of the MKS complex genes, *tmem-231* (MIM 614949), *mks-1* (MIM 609883), *mksr-1* (the ortholog of *B9d1* [MEM 614144]), *mksr-2* (the ortholog of *B9d2* [MIM 611951]), *mks-3* (the ortholog of *Tmem67* [609884]), and *mks-6* (the ortholog of *Cc2d2a* [MIM 612013]), as well as the orthologs of the NPHP complex genes *nphp-1* (MIM 607100) and *nphp-4* (MIM 607215), encode transition zone proteins [13, 22–26]. Individual loss of function of these genes does not dramatically compromise *C*. *elegans* ciliary structure. However, loss of function of one of several MKS complex genes when combined with loss of function of either *nphp-1* or *nphp-4* synergistically compromises ciliary structure in *C*. *elegans* [13, 22, 23].

Like MKS and NPHP complex proteins, many proteins associated with BBS form a large complex, the BBSome [27]. The BBSome functions as a vesicular coat that transports select G protein-coupled receptors (GPCRs) to the cilium through an association with intraflagellar transport (IFT) machinery [28, 29]. In contrast to MKS or NPHP components, abrogating the function of individual BBS components in *C*. *elegans* disrupts ciliary structure [30]. Whether the BBSome works together with the transition zone is unknown. However, mutation in *MKS1* can cause BBS, and mutation in *BBS4* (MIM 600374) can modify the ciliopathy phenotypes of *CEP290* (MIM 610142), suggesting that the BBSome and transition zone may share some functions [31, 32].

We hypothesized that genetic interactions between MKS, NPHP, and BBS complex genes could contribute to the wide phenotypic spectrum observed in human ciliopathies. To test this hypothesis, we generated animals with intra- and inter-complex double mutations, with an emphasis on the ciliopathy gene *Tectonic1* (*Tctn1* [MIM 609863]). We previously found that Tctn1 is an essential component of the transition zone required for cilium-dependent Hh signaling in mice, and that human *TCTN1* is mutated in another ciliopathy, Joubert syndrome [16, 33]. Here, we identify the *C*. *elegans* ortholog of *Tctn1*, *tctn-1*, and find that it also encodes a transition zone protein. Loss of TCTN-1 does not disrupt *C*. *elegans* ciliary structure, similar to other transition zone genes. *tctn-1* interacts with *nphp-4* and *nphp-1*, but not with the MKS complex genes *mks-1*, *mksr-1*, *mksr-2* and *mks-3*, thereby genetically placing *tctn-1* within the MKS module. These genetic interactions in nematodes predicted mammalian genetic interactions, as combining mouse *Tctn1* and *Nphp4* mutations also resulted in synthetic ciliary phenotypes. Additional double mutations between one gene of the MKS complex and one gene of the NPHP complex yielded similar synthetic effects, suggesting that the genetic interactions between MKS and NPHP complex components may pertain to many members. In contrast, double mouse mutations between genes within the same complex did not show such an interaction, consistent with findings in *C*. *elegans*. Furthermore, both *C*. *elegans* and mammals exhibited a genetic synergy between mutations affecting the transition zone and the BBSome, indicating that the MKS, NPHP, and BBS complexes share overlapping functions in ciliogenesis. Hence, mutations affecting distinct ciliopathy protein complexes synthetically interact to modify cilia-associated phenotypes.

## Results

### *C*. *elegans* Tectonic is a transition zone component

**Fig 1:**
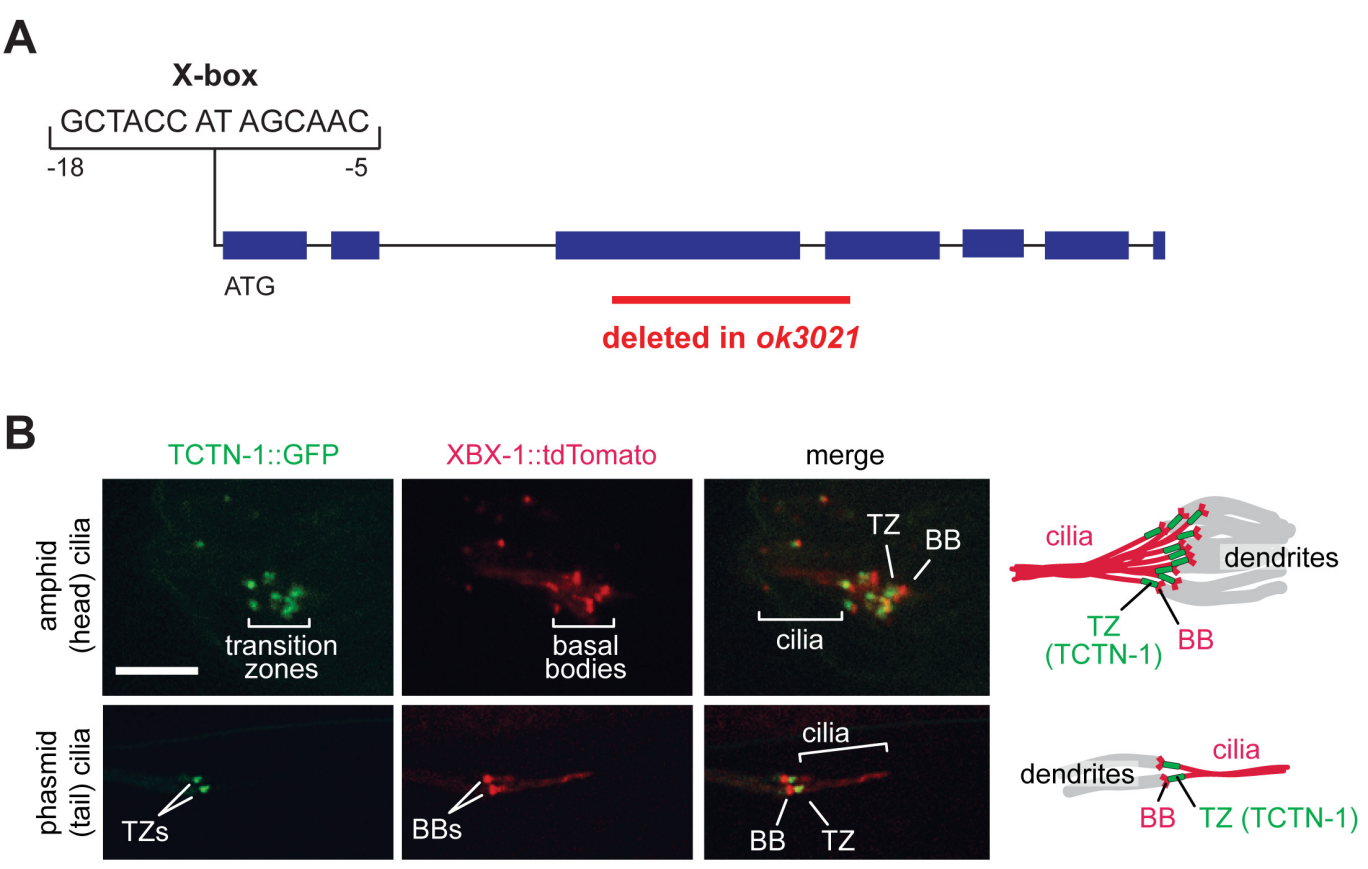
The *C*. *elegans* ortholog of Tectonic1 is a transition zone component. (A) Schematic of the X-box sequence and *ok3021* allele of *C*. *elegans tctn-1*. (B) GFP-tagged TCTN-1 localizes specifically to the transition zones (TZs) in head (amphid) and tail (phasmid) cilia of *C*. *elegans*. Basal bodies (BBs) and ciliary axonemes are marked with tdTomato-tagged XBX-1, a component of the ciliary dynein. Schematics illustrate the position of TCTN-1 at the transition zone with respect to the basal body and axoneme. Scale bar, 5 μm.

By sequence homology, we identified a single *C*. *elegans* ortholog of the three vertebrate *Tectonic* genes, *E04A4*.*6*, which we refer to as *tctn-1*. Mammalian Tectonics possess a signal peptide and a domain of unknown function (DUF1619) characteristic of the family. *C*. *elegans tctn-1* also possesses a signal peptide and is most homologous to other Tectonics in the C-terminal region (S1 Fig). In nematodes, ciliary genes often contain a conserved X-box sequence that acts as a binding site for the ciliogenic RFX transcription factor, DAF-19 [34]. Consistent with a ciliary function, *tctn-1* has a predicted X-box sequence (Fig 1A), is strongly downregulated in *daf-19* mutants, and is preferentially expressed in ciliated sensory neurons [35–37]. For these reasons, we surmised that *tctn-1* was likely to have a ciliary function in *C*. *elegans*.

To investigate whether nematode TCTN-1 localizes to cilia, we generated a strain that expresses a carboxy-terminal GFP-tagged version of TCTN-1 under a *bbs-8* promoter active in ciliated cells [38]. To visualize cilia, we marked basal bodies and axonemes with a tdTomato-tagged ciliary dynein light intermediate chain, XBX-1. TCTN-1-GFP was enriched specifically at the transition zone of cilia, immediately distal to the basal body and proximal to the axoneme (Fig 1B).

### *tctn-1* genetically interacts with NPHP complex genes, but not MKS complex genes

To investigate the function of TCTN-1, we obtained the *E04A4*.*6*(*ok3021*) mutant from the *C*. *elegans* Gene Knockout Consortium. The *ok3021* allele contains a 515 base pair deletion spanning exons three and four of *tctn-1*, which generates a premature stop codon and is predicted to disrupt the function of *tctn-1* (Fig 1A).

**Fig 2:**
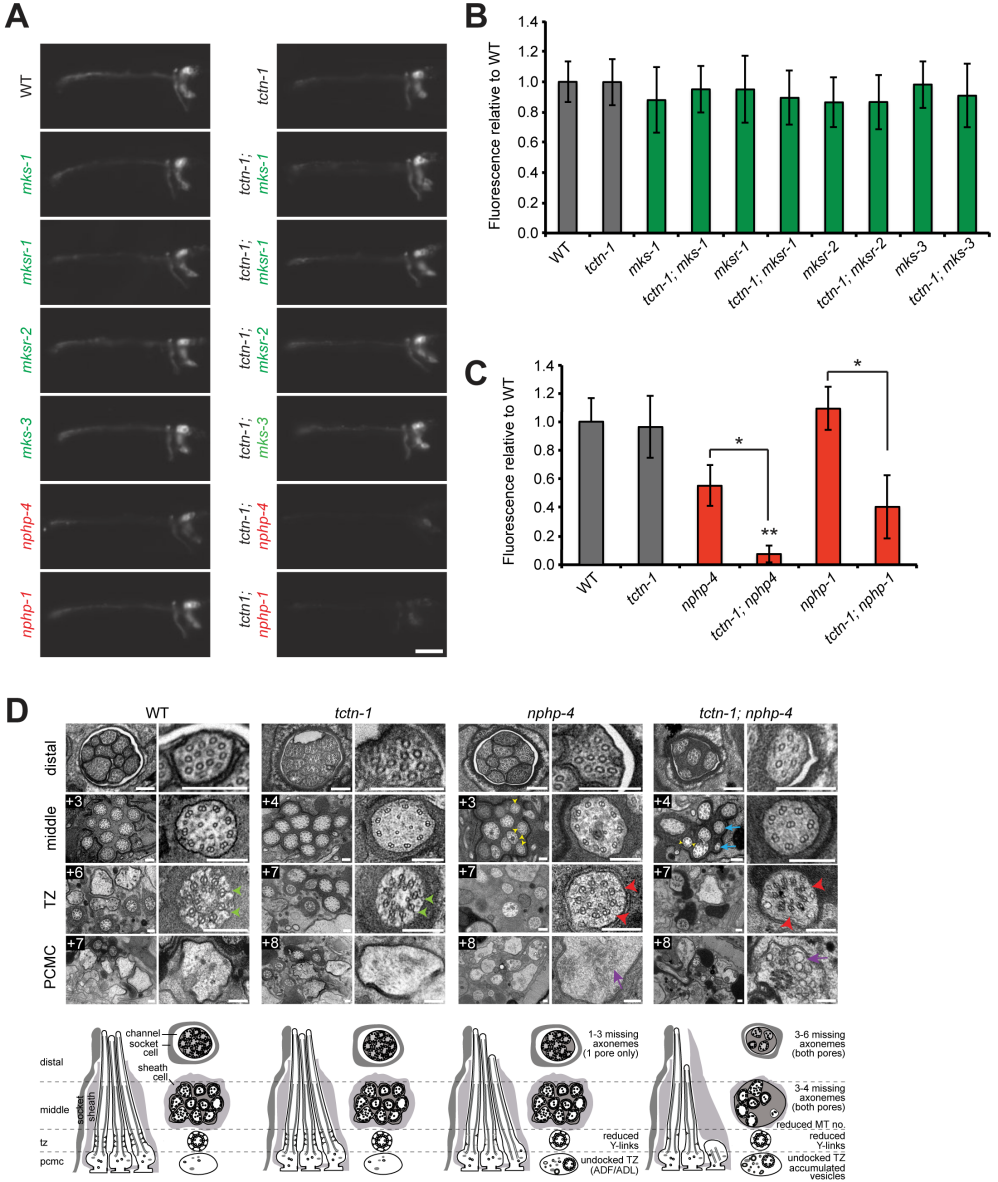
*C*. *elegans tctn-1* interacts with NPHP genes, but not MKS genes to affect ciliary structure. (A) Dye filling of amphid neurons in L4 nematodes in the indicated single transition zone mutants (left column) and *tctn-1* double mutants (right column). Lateral views, with anterior to the left. Genotypes including an allele affecting a previously recognized MKS complex component are indicated in green. Genotypes including an allele affecting an NPHP complex component are indicated in red. Scale bar, 20 μm. (B and C) Fluorescence intensity of DiI filled amphid neurons in single mutants of the MKS complex or NPHP complex, and double mutants with *tctn-1* relative to wild type. Error bars represent the standard deviation. Statistical significance according to unpaired Student’s *t*-tests (* *p*<0.001; ** *p*<0.001 compared to *tctn-1*). (D) Low and high magnification TEM cross-sections of the distal segment, middle segment, transition zone (TZ), and distal dendritic periciliary membrane compartment (PCMC) of amphid cilia with schematics below (lateral and transverse views). Green arrowheads indicate intact Y-links in wild type and *tctn-1* transition zones whereas red arrowheads indicate reduced or missing Y-links, observed in *nphp-4* and *tctn-1; nphp-4* transition zones. Yellow arrowheads indicate open B-tubules and purple arrows indicate vesicle accumulation in the PCMCs of *nphp-4* and *tctn-1; nphp-4* mutants. *tctn-1; nphp-4* mutants display several truncated axonemes and axonemes with fewer microtubule doublets (blue arrows) compared to wild type, *tctn-1* and *nphp-4* mutants. Boxed numbers indicate distances (μm) from the distal ciliary tips. Scale bars,100 nm.

Homozygous *tctn-1* mutants exhibited no alteration of growth, size, egg laying, brood size, or cilium-associated sensory behaviors (osmotic avoidance or chemotaxis to diacetyl or butanone) (S2 Fig). Additionally, the sensory neurons of *tctn-1* mutants were indistinguishable from those of the wild type N2 reference strain in their ability to take up the hydrophobic dye, DiI. This dye filling assay is a simple method of indirectly testing the structural integrity of cilia of six head (amphid) neurons and two tail (phasmid) neurons [39], and suggests that ciliary structure is not grossly compromised in the absence of TCTN-1 (Fig 2A–2C). Other *C*. *elegans* transition zone gene mutants such as *nphp-1*, *nphp-4*, *mks-1*, *mksr-1*, *mksr-2*, and *mks-3* mutants, similarly possess largely normal ciliary structure (Fig 2A–2C) [13, 22–24].

Previous studies have found that a mutation in either of the *C*. *elegans* homologs of the NPHP complex components *nphp-1* or *nphp-4*, combined with a mutation in an MKS complex gene, synergistically disrupts ciliary structure and consequently causes a dye filling defect [13, 22, 23]. To test whether *tctn-1* functions as an MKS complex or NPHP complex gene, we generated double mutants of *tctn-1* and either MKS complex genes or NPHP complex genes. Double mutations affecting *tctn-1* and any of four MKS complex genes (*tctn-1; mks-1*, *tctn-1; mksr-1*, *tctn-1; mksr-2*, *and tctn-1; mks-3*) did not alter dye filling, similar to *tctn-1* single mutants (Fig 2A and 2B).

In contrast, dye filling in *tctn-1; nphp-4* double mutants was dramatically disrupted compared to *tctn-1* or *nphp-4* single mutants. In *tctn-1; nphp-4* double mutants, amphid neurons incorporated dye at low or undetectable levels, resulting in decreased fluorescence relative to either single mutant (Fig 2A and 2C). Similarly, *tctn-1; nphp-1* double mutants were unable to incorporate dye compared to either single mutant (Fig 2A and 2C). Therefore, *tctn-1* synergistically disrupts ciliary structure when combined with NPHP but not MKS complex mutations, genetically placing *tctn-1* within the MKS complex.

### TCTN-1 and NPHP-4 have overlapping functions in supporting ciliary structure

The inability of the sensory neurons of *tctn-1; nphp-4* mutants to dye fill suggested that their ciliary structure or morphology was defective. *tctn-1; nphp-4* phasmid cilia, visualized by expressing the fluorescently tagged ciliary protein XBX-1::tdTomato, were shorter than cilia of wild type or either single mutant (S3A and S3B Fig). Additionally, many *tctn-1; nphp-4* cilia were mispositioned; for example, they were frequently abnormally close to the cell body at the ends of short dendrites, or not projecting posteriorly (S3C and S3D Fig). These perturbations in ciliary position are similar to those caused by concurrent loss of NPHP-4 and other MKS complex components [13, 22, 23].

To gain further insight into how TCTN-1 and NPHP-4 cooperatively support ciliogenesis, we examined the amphid channel cilia of *tctn-1* and *nphp-4* single mutants, and *tctn-1; nphp-4* double mutants using transmission electron microscopy. In *tctn-1* single mutants, the ten cilia of both amphid channel pores were indistinguishable from wild type. Notably, the ciliary transition zones of *tctn-1* mutants contained intact Y-links and exhibited no overt abnormalities, suggesting that TCTN-1 is not essential for generating all structural components of the transition zone (Fig 2D).

In contrast, *nphp-4* single mutants displayed cilia with moderate ultrastructural defects. In addition to the modest shortening of 1–3 axonemes in one amphid pore (the other pore is normal) and the occurrence of open B-tubules in the middle segment previously described [25], some *nphp-4* mutant cilia exhibited a small accumulation of vesicles in the periciliary membrane compartment (PCMC) (Fig 2D). We also detected defects in the transition zone of *nphp-4* mutants (Lambacher *et al*., manuscript in revision). Y-links, the structural hallmark of the transition zone, were frequently reduced in number and appeared less electron dense in *nphp-4* mutants. Despite the reduction in Y-links, the ciliary membrane remained closely opposed to the axoneme in most *nphp-4* transition zones. The only exceptions were the transition zones of the biciliated ADF and ADL neurons; in *nphp-4* mutants, these transition zones were frequently fully disconnected from the ciliary membrane and positioned ectopically in the distal dendrite region (Fig 2D).

The ciliary structure of *tctn-1; nphp-4* double mutants was more severely disrupted than that of *nphp-4* single mutants. In *tctn-1; nphp-4* mutants, at least 3–4 axonemes were missing in the middle and distal regions of both amphid pores, and remaining axonemes frequently possessed fewer microtubule doublets (Fig 2D). Additionally, *tctn-1; nphp-4* mutant cilia displayed defects in Y-links at the transition zone similar to *nphp-4* mutants: Y-links were either thinner than wild type or missing, and in some cases, transition zone microtubules were completely disconnected from the ciliary membrane (Fig 2D). An accumulation of membranes and vesicles was also observed in the PCMC of *tctn-1; nphp-4* mutants. In contrast, *tctn-1; mks-3* double mutants displayed no abnormalities in ciliary ultrastructure (S3E Fig), consistent with the lack of genetic interaction between *tctn-1* and other MKS complex components. Thus, we conclude that ciliary structure is dependent on the overlapping functions of TCTN-1 and NPHP-4.

### *C*. *elegans* TCTN-1 contributes to the ciliary gate function, but not to transition zone protein composition

**Fig 3:**
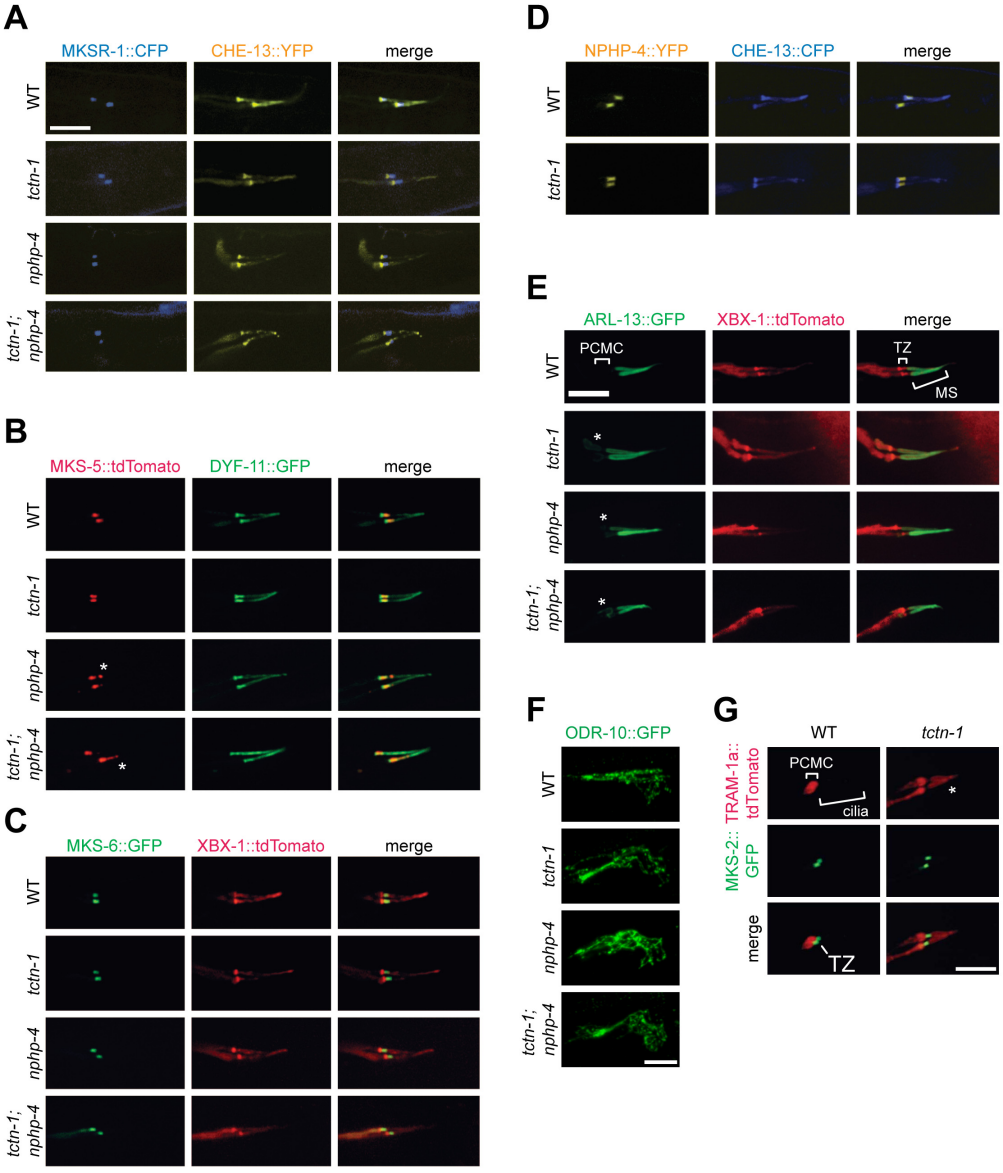
TCTN-1 contributes to *C*. *elegans* ciliary gate function. (A-D) Localization of different transition zone proteins in phasmid cilia of wild type, *tctn-1*, *nphp-4*, and *tctn-1; nphp-4* mutants. Each GFP, CFP, YFP, or tdTomato-tagged transition zone protein is co-localized with a ciliary marker (CHE-13, DYF-11 or XBX-1), as indicated. Abnormal localization of MKS-5 in the *nphp-4* single and *tctn-1; nphp-4* double mutants is indicated by asterisks. (E) ARL-13::GFP localizes ectopically to the PCMC in *tctn-1*, *nphp-4*, and *tctn-1; nphp-4* mutants as indicated by asterisks, but localizes normally to the middle segment (MS) of cilia. (F) ODR-10::GFP localizes to the cilia of AWA neurons in wild type, *tctn-1*, *nphp-4*, and *tctn-1; nphp-4* mutants. Anterior is to the right. (G) TRAM-1a::tdTomato localizes only to the PCMC in wild type animals, but enters phasmid cilia in *tctn-1* mutants, as indicated by asterisks. MKS-2::GFP marks the transition zone. Scale bars, 5 μm.

We hypothesized that disruption of Y-links in *nphp-4* single mutants and *tctn-1; nphp-4* double mutants could reflect an abnormal composition of the transition zone. Using established markers of the ciliary base and axoneme, we assessed the localization of several fluorescently tagged transition zone proteins. MKSR-1, MKS-5, MKS-6 and NPHP-4 all localized to the transition zone of *tctn-1* mutants in a manner indistinguishable from wild type (Fig 3A–3D). Similarly, despite the reduction in Y-links identified in *nphp-4* and *tctn-1; nphp-4* mutants, MKSR-1, MKS-6 and NPHP-4 localized to the transition zone of these mutants equivalently to wild type (Fig 3A, 3C and 3D). MKS-5 also localized to the transition zone of *nphp-4* and *tctn-1; nphp-4* mutants, although it partially mislocalized distally along the ciliary axoneme (Fig 3B).

The transition zone is proposed to function as a ciliary gate, controlling protein entry, retention, and exclusion [12–14, 16, 40]. The small GTPase, ARL-13 and its ortholog Arl13b localize to cilia in *C*. *elegans* and vertebrates, respectively [41, 42]. In *C*.*elegans*, transition zone genes including *nphp-4* are required for ARL-13 to exclusively localize to cilia [43]. To test if *C*. *elegans* TCTN-1 regulates the localization of ARL-13, we examined a fusion of ARL-13 and GFP. As in wild type animals, in *tctn-1* single and *tctn-1; nphp-4* double mutants, ARL-13::GFP localized along the length of the axoneme. However, unlike wild type animals, *tctn-1* and *tctn-1; nphp-4* mutants displayed a modest accumulation of ARL-13::GFP in the PCMC (Fig 3E). Thus, similar to other *C*. *elegans* transition zone genes, TCTN-1 is not required for ARL-13::GFP to enter the cilium, but may be required for its retention within the cilium [43].

In addition to ARL-13, signaling proteins such as GPCRs can localize to cilia. To test if TCTN-1 regulates the ciliary localization of GPCRs, we examined whether it was required for the ciliary localization of ODR-10, a GPCR that senses the odorant diacetyl at the cilia of AWA neurons [44, 45]. ODR-10::GFP localized to AWA cilia equivalently in wild type and *tctn-1*, *nphp-4*, and *tctn-1; nphp-4* mutants (Fig 3F), consistent with our finding that none of these genes is required for the behavioral responses to diacetyl (S2F Fig). Thus, *C*. *elegans* TCTN-1 is not essential for the ciliary localization of a GPCR.

In addition to promoting the localization of ciliary proteins, the transition zone is proposed to exclude non-ciliary proteins from entering the cilium. To test whether TCTN-1 participates in this aspect of transition zone function, we examined the localization of TRAM-1a, a transmembrane protein excluded from the cilium [13]. In wild type animals, TRAM-1a::tdTomato was confined to a ring of periciliary membrane at the distal end of the dendrite and was not observed in the ciliary axoneme. In contrast, TRAM-1a::tdTomato was present within the cilia of *tctn-1* mutants (Fig 3G). Together, these findings indicate that *C*. *elegans* TCTN-1 is not essential for MKS or NPHP protein complex localization to the transition zone, nor for the ciliary localization of ARL-13::GFP or ODR-10::GFP, but is required to exclude a non-ciliary protein from the cilium. Therefore, TCTN-1 may be a key component of the ciliary barrier preventing the entry of non-ciliary proteins

### The NPHP complex and BBS-5 have overlapping functions in *C*. *elegans* ciliogenesis

The genetic interaction between *tctn-1* and NPHP complex genes strengthens previous indications that the MKS and NPHP complexes perform overlapping functions within the transition zone [13, 15, 22, 23]. We investigated whether a biochemical complex that does not specifically localize to the transition zone, the IFT-associated BBSome complex, could also have overlapping functions with transition zone complexes. Core BBSome components, BBS-1, BBS-2, BBS-4, BBS-5, BBS-7, BBS-8, and BBS-9 are conserved in *C*. *elegans*. Except for *bbs-5* (MIM 603650) mutants, neurons of *C*. *elegans bbs* mutants do not incorporate dye, thus precluding the use of dye filling to analyze synthetic interactions with most BBS-associated genes [30, 46]. Although the *bbs-5(gk507)* allele contains a 680 bp deletion spanning the first two exons of the *bbs-5* gene, neurons of homozygous *bbs-5(gk507)* mutants dye fill normally [46, 47], suggesting that this allele is hypomorphic or that BBS-5 is more dispensable for BBSome function than other components.

**Fig 4:**
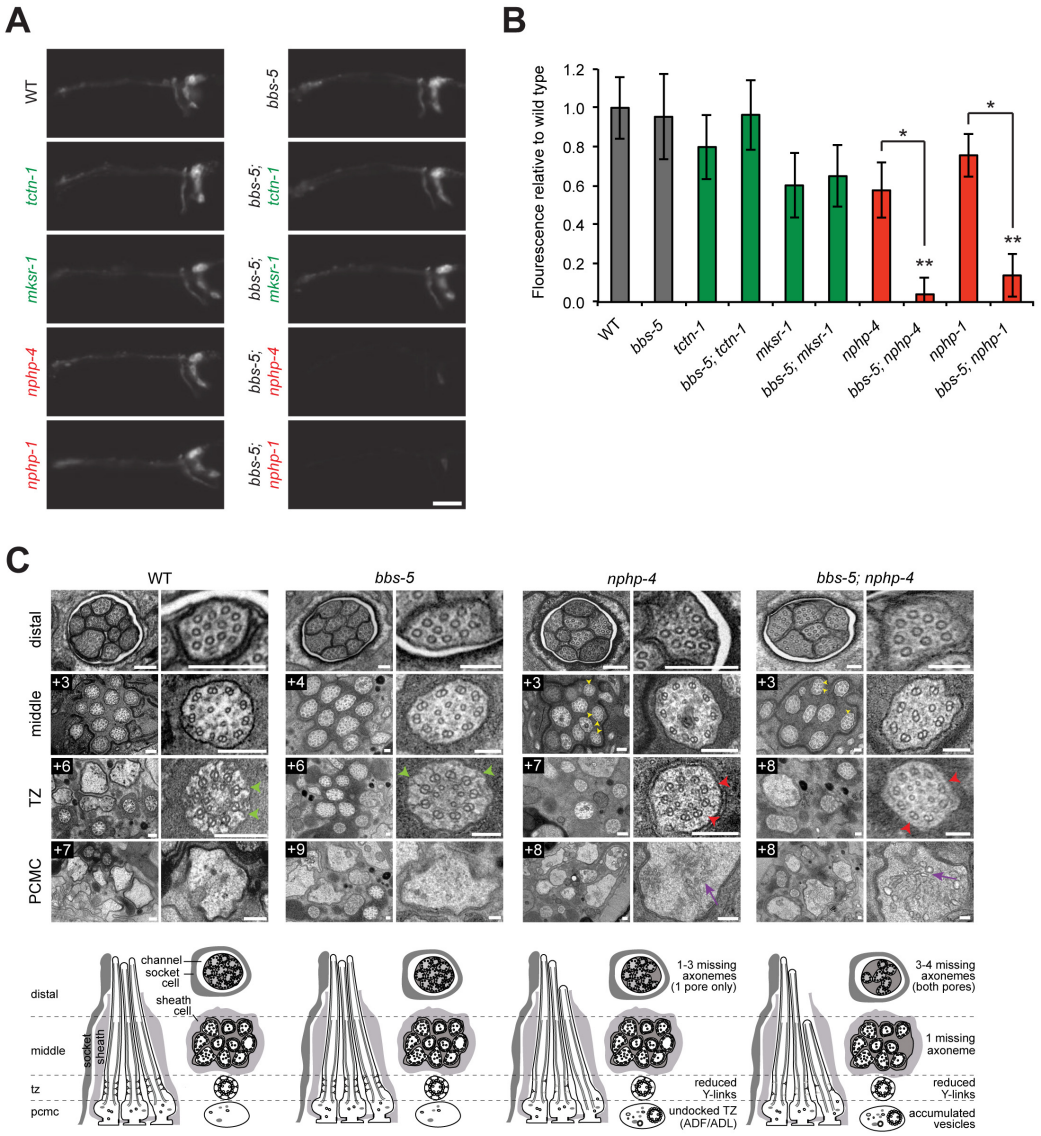
*C*. *elegans* NPHP genes synthetically interact with *bbs-5* to affect ciliary structure. (A) Dye filling of amphid neurons in L4 nematodes in single transition zone mutants (left column) and *bbs-5* double mutants (right column). Lateral views, with anterior to the left. Scale bar, 20 μm. (B) Fluorescence intensity of DiI filled amphid neurons relative to wild type. Genotypes including an allele affecting an MKS complex component are indicated in green. Genotypes including an allele affecting an NPHP complex component are indicated in red. Error bars represent the standard deviation. Statistical significance according to unpaired Student’s *t*-tests (* *p*<0.001; ** *p*<0.001 compared to *bbs-5*). (C) Low and high magnification TEM cross-sections of the distal segment, middle segment, transition zone (TZ), and PCMC of amphid cilia with schematics below (lateral and transverse views). *bbs-5* mutants display normal ciliary structures, including intact Y-links (green arrowheads). *bbs-5; nphp-4* mutant cilia display open B-tubules (yellow arrowheads), reduced Y-links (red arrowheads) and vesicle accumulation in the PCMC (purple arrows) similar to *nphp-4* cilia, but have fewer axonemes that fully extend distally in both pores. Boxed numbers indicate distances (μm) from the distal ciliary tips. Scale bars,100 nm.

To test for genetic interactions between *bbs-5* and transition zone genes, we examined dye filling in double mutants for *bbs-5* and MKS complex genes or NPHP complex genes. *bbs-5; tctn-1* and *bbs-5; mksr-1* double mutants dye filled to the same extent as their single mutant counterparts, revealing no genetic interaction between *bbs-5* and MKS complex genes (Fig 4A and 4B). In contrast, dye filling in *bbs-5; nphp-4* and *bbs-5; nphp-1* double mutants was dramatically decreased compared to their respective single mutants (Fig 4A and 4B). Therefore, BBS-5 and NPHP complex components have overlapping functions in supporting *C*. *elegans* ciliary integrity. The finding that *bbs-5* synthetically interacts with NPHP complex genes but not MKS complex genes further suggests that the NPHP and MKS complexes perform distinct roles.

To further investigate the interaction between *bbs-5* and NPHP genes, we examined *bbs-5; nphp-4* double mutant cilia by transmission electron microscopy. As predicted by the dye filling assay, *bbs-5* mutants showed normal amphid channel cilium ultrastructure, with a full complement of ten axonemes extending through the length of each pore (Fig 4C). Moreover, the transition zones of *bbs-5* mutants contained normal Y-links, and the ciliary membrane and microtubule core were indistinguishable from those of wild type animals (Fig 4C).

*bbs-5; nphp-4* double mutant cilia displayed more pronounced ultrastructural defects than *bbs-5* or *nphp-4* single mutants. Similar to *nphp-4* cilia, *bbs-5; nphp-4* cilia possessed open B-tubules, a reduction in Y-links and vesicle accumulation in some PCMCs (Fig 4C). In contrast to the single mutants, *bbs-5; nphp-4* double mutants were missing 3–4 axonemes in the distal region and one axoneme in the middle region of both amphid pores (Fig 4C). Thus, BBS-5 has overlapping functions with the NPHP complex in building or maintaining the ciliary axoneme.

### Murine MKS and NPHP complexes have overlapping functions in limb development

Mutations in human *NPHP4* cause nephronophthisis, either with or without retinal degeneration [48, 49]. To test whether the genetic interactions detected in *C*. *elegans* with MKS complex genes could contribute to the expressivity of *NPHP4*, we investigated whether the same genetic interactions occur in mammals. *Nphp4* ^nmf192/nmf192^ mutant mice (hereinafter referred to as *Nphp4* ^n/n^) are viable, but develop retinal degeneration and have reduced sperm motility [50]. Mouse *Tctn1* ^-/-^ mutants die during late gestation and exhibit heterotaxia, microphthalmia, and hindlimb polydactyly, phenotypes associated with ciliary malfunction and similar to those exhibited by human MKS affected individuals [16, 33]. Several other mouse MKS complex mutants, such as *Tctn2* ^-/-^ (MIM 613846) and *Cc2d2a* ^-/-^ (also known as *Mks6*), display highly similar phenotypes [16, 21].

**Fig 5:**
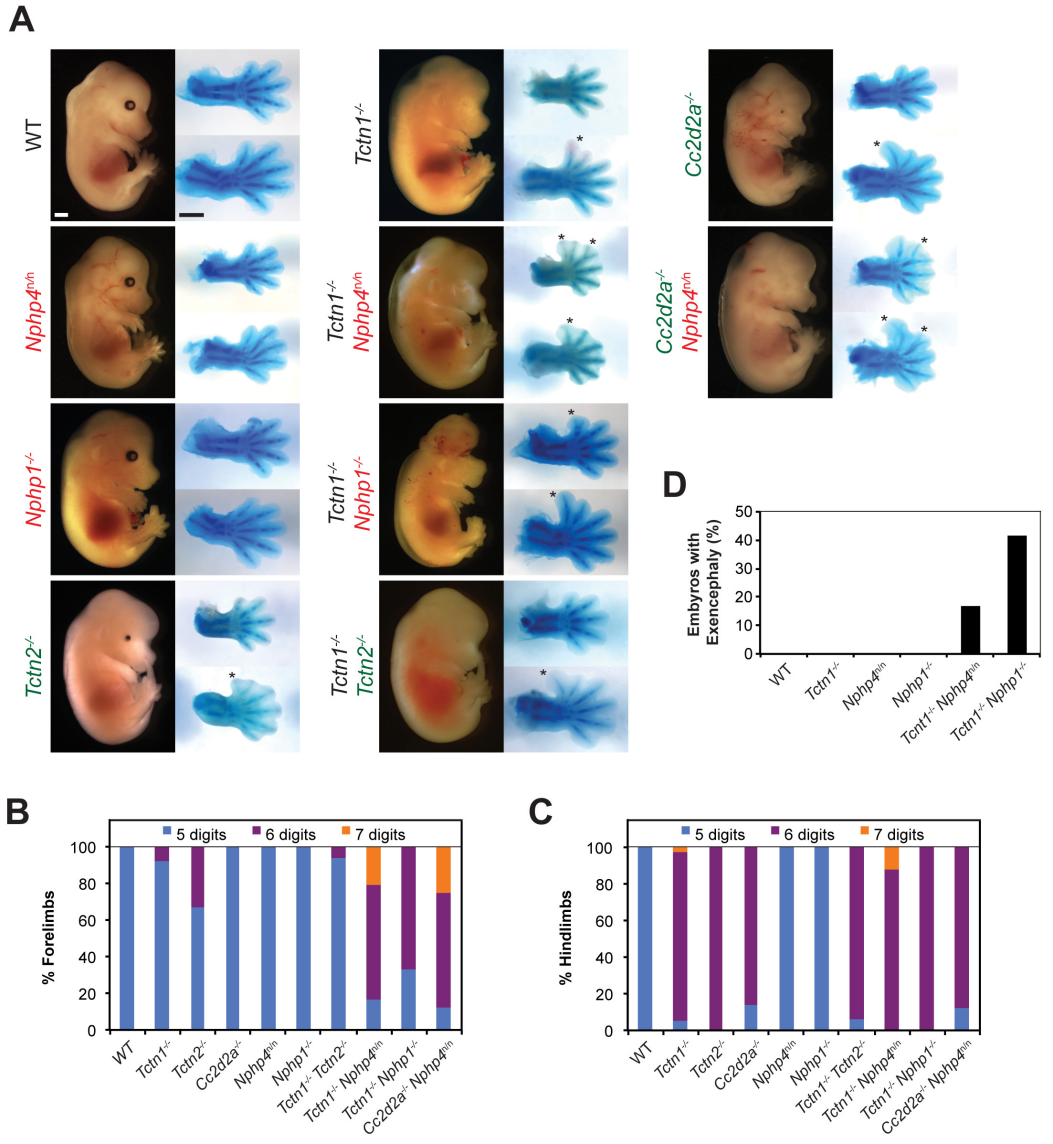
Mouse *Tctn1* genetically interacts with NPHP complex genes, but not MKS complex genes. (A) Lateral views of wild type, single or double mutant mouse embryos of indicated genotype at E14.5. Exencephaly is apparent in the *Tctn1* ^-/-^ *Nphp1* ^-/-^ double mutant. Corresponding Alcian blue staining of the right forelimb (top) and hindlimb (bottom) are included, with asterisks indicating extra digits. Genes encoding components of the NPHP complex are indicated in red. Genes encoding components of the MKS complex are indicated in green. Scale bars, 1 mm. (B) Number of digits in the forelimbs and (C) hindlimbs of wild type, single or double mutant embryos of indicated genotypes. (D) Incidence of exencephaly in wild type, single or double mutants embryos of indicated genotypes. Numbers of animals analyzed for polydactyly and exencephaly are included in S1 and S2 Tables.

To assess potential genetic interactions between the MKS and NPHP complexes in mammals, we generated mice doubly mutant for *Tctn1* and *Nphp4*. *Tctn1* ^-/-^ mutants exhibited single digit polydactyly restricted to the hindlimbs and *Nphp4* ^n/n^ mutants had no limb abnormalities. In contrast, *Tctn1* ^-/-^ *Nphp4* ^n/n^ double mutants exhibited polydactyly in both the forelimb and hindlimb, and had an increased number of digits per limb compared to the polydactyly of *Tctn1* ^-/-^ mutants (Fig 5A–5C, S1 Table). In addition, *Tctn1* ^-/-^ *Nphp4* ^n/n^ double mutants exhibited partially penetrant exencephaly, which was not observed in *Tctn1* ^-/-^ single mutants or in *Nphp4* ^n/n^ single mutants (Fig 5D, S2 Table). Therefore, *Tctn1* and *Nphp4* interact synergistically in mammals, as they do in *C*. *elegans*.

Like *NPHP4*, human mutations in *NPHP1* are associated with multiple ciliopathies, including nephronophthisis and Joubert syndrome, characterized by cerebellar vermis hypoplasia [51–53]. To determine if the genetic interaction between *Tctn1* and *Nphp4* was specific to *Nphp4* or extended to another NPHP complex gene, we generated *Tctn1* ^-/-^ *Nphp1* ^-/-^ double mutant mice. As with *Tctn1* ^-/-^ *Nphp4* ^n/n^ double mutants, *Tctn1* ^-/-^ *Nphp1* ^-/-^ double mutants displayed more severe polydactyly and a higher penetrance of exencephaly compared to *Tctn1* ^-/-^ mutants (Fig 5A–5D, S1 and S2 Tables). These results reveal that in both nematodes and mice, *Tctn1* displays a synthetic genetic interaction with both *Nphp1* and *Nphp4*.

To assess whether the interaction of NPHP complex genes with *Tctn1* extended to other MKS complex genes, we generated double mutants affecting *Nphp4* and *Cc2d2a*. In addition to MKS, human *CC2D2A* mutations are associated with Joubert syndrome [54–56]. Like *Tctn1* ^-/-^ mutants, mouse *Cc2d2a* ^-/-^ mutants displayed single digit polydactyly restricted to the hindlimb [16]. Similar to *Tctn1* ^-/-^ *Nphp4* ^n/n^ double mutants, *Cc2d2a* ^-/-^ *Nphp4* ^n/n^ double mutants displayed polydactyly in the forelimbs unlike either single mutant (Fig 5A–5C, S1 Table). Therefore, the genetic interaction between the MKS and NPHP complexes extends to multiple genes of both complexes.

As mutations within the MKS complex or within the NPHP complex do not genetically interact when combined in *C*. *elegans*, we tested whether this principle held true in mice. We generated *Tctn1* ^-/-^ *Tctn2* ^-/-^ double mutant mice and found that they did not phenotypically differ from *Tctn1* ^-/-^ or *Tctn2* ^-/-^ single mutants (Fig 5A–5C, S1 Table). Similarly, we generated *Nphp1* ^-/-^ *Nphp4* ^n/n^ double mutant mice, which grow into adulthood at rates comparable to control littermates (S4A Fig). Thus, at a gross level in both nematodes and mammals, multiple mutations affecting different MKS complex components or multiple mutations affecting different NPHP complex components do not additively disrupt ciliary function. These results further suggest that the MKS complex and the NPHP complex do not possess residual function in the absence of individual components.

### Murine Tctn1 and Nphp4 cooperatively support ciliogenesis

**Fig 6:**
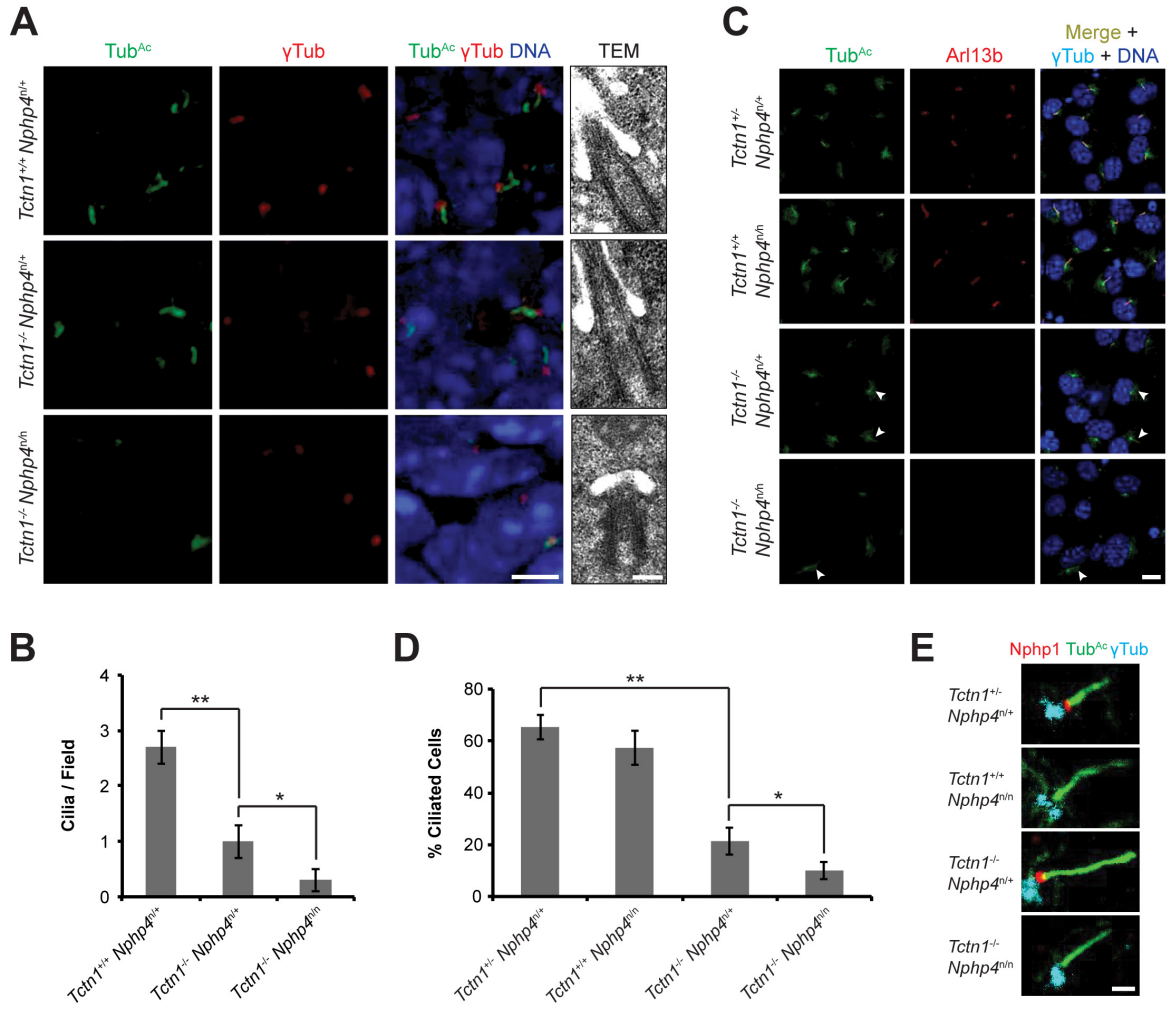
Mouse Tctn1 and Nphp4 have distinct roles in transition zone composition and overlapping roles in ciliogenesis. (A) Limb bud sections (left) from E11.5 embryos were stained for acetylated tubulin (Tub^Ac^, green) to mark the ciliary axonemes, γ-tubulin (red) to mark the basal bodies and DAPI (blue) to mark nuclei. Scale bar, 5 μm. Transmission electron microscopy (TEM, right) for each genotype. Scale bar, 200 nm. (B) Quantification of limb bud cilia in control, *Tctn1* ^-/-^ *Nphp4* ^n/+^, and *Tctn1* ^-/-^ *Nphp4* ^n/n^ mutants from TEM fields of view. Error bars represent the standard error of the mean. Statistical significance according to unpaired Student’s *t*-tests (* *p*<0.05; ** *p*<0.001). (C) Fibroblasts derived from E13.5 limb buds of the indicated genotypes stained for Tub^Ac^ (green), Arl13b (red), γ-tubulin (cyan), and DAPI (blue). White arrowheads indicate cilia in *Tctn1* ^-/-^ *Nphp4* ^n/+^ and *Tctn1* ^-/-^ *Nphp4* ^n/n^ mutants. Scale bar, 10 μm. (D) Quantification of proportion of ciliated cultured limb bud fibroblasts. Error bars represent the standard deviation. Statistical significance according to unpaired Student’s *t*-tests (* *p*<0.05; ** *p*<0.0001). (E) Immunostaining of limb bud fibroblasts for Nphp1 (red), Tub^Ac^ (green), and γ-tubulin (cyan). Scale bar, 1 μm.

The genetic interaction of *Tctn1* and *Nphp4* suggested that the mammalian MKS and NPHP complexes have partially overlapping ciliary functions. To investigate how these two complexes participate in ciliary functions, we examined cilia in the forelimb, a tissue phenotypically affected in *Tctn1* ^*-/-*^ *Nphp4* ^n/n^ double mutants. Immunostaining revealed that ciliation in *Tctn1* ^*-/-*^ forelimbs was reduced compared to controls (Fig 6A). *Tctn1* ^-/-^ *Nphp4* ^n/n^ forelimbs were further depleted of cilia compared to *Tctn1* ^-/-^ forelimbs (Fig 6A). Transmission electron microscopy confirmed the depletion of cilia in the forelimb buds of *Tctn1* ^-/-^ *Nphp4* ^n/n^ double mutants, which possessed docked basal bodies, but lacked ciliary axonemes (Fig 6A and 6B). The greater ciliogenesis defect in *Tctn1* ^-/-^ *Nphp4* ^n/n^ forelimbs likely accounts for the increased polydactyly, as cilia are required for Shh-dependent patterning of the limb buds [57–59].

To further investigate how Tctn1 and Nphp4 participate in ciliary functions, we cultured fibroblasts derived from mutant and control limb buds. Limb bud fibroblasts showed similar degrees of ciliation in vitro as they did in vivo (Fig 6C and 6D). Although the majority of *Tctn1* ^-/-^ *Nphp4* ^n/n^ mutant limb fibroblasts lacked cilia, a small percentage of cells remained ciliated. In the ciliated *Tctn1* ^-/-^ *Nphp4* ^n/n^ mutant cells, we investigated whether ciliary or transition zone protein composition was disturbed. The small GTPase Arl13b localized to cilia in control and *Nphp4* ^n/n^ mutant fibroblasts, but was absent in *Tctn1* ^-/-^ and *Tctn1* ^-/-^ *Nphp4* ^n/n^ mutant cilia (Fig 6C). Interestingly, *C*. *elegans* TCTN-1 was dispensable for much of the cellular ARL-13:GFP to localize to cilia (Fig 3E), demonstrating a difference in the function of *C*. *elegans* and mouse Tctn1.

We examined the transition zone localization of Nphp1, a member of the NPHP complex. In control and *Tctn1* ^-/-^ mutant fibroblasts, Nphp1 localized to the transition zone, but in *Nphp4* ^n/n^ single and *Tctn1* ^-/-^ *Nphp4* ^n/n^ double mutant fibroblasts, Nphp1 was absent from the transition zone (Fig 6E). Hence, mouse Nphp4 is required for the localization of Nphp1, as it is in *C*. *elegans* [24]. Therefore, in mice, ciliary localization of Arl13b depends specifically on Tctn1, and not on Nphp4, whereas transition zone localization of Nphp1 depends specifically on Nphp4, and not on Tctn1. Thus, Tctn1 and Nphp4 have overlapping functions in promoting ciliogenesis, and distinct roles in controlling ciliary protein localization.

**Fig 7:**
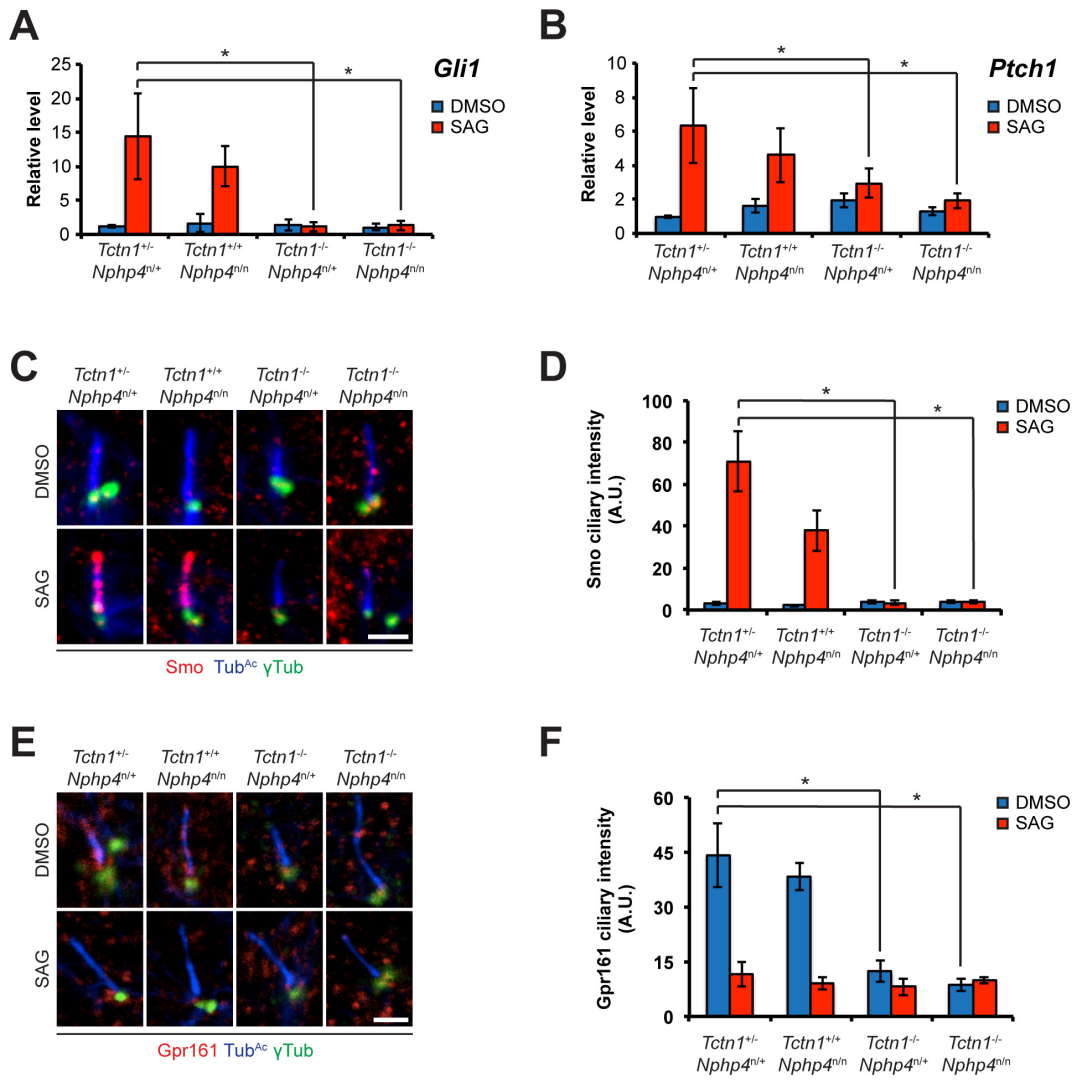
Roles for mouse *Tctn1* and *Nphp4* in Hh signaling and ciliary localization of Hh pathway components. (A and B). mRNA levels of *Gli1* and *Ptch1* normalized to *β-actin* in forelimb bud cells treated with DMSO or SAG. Error bars represent standard deviations. Statistical significance according to unpaired Student’s *t*-tests (* *p*<0.05). (C) Limb bud fibroblasts treated with DMSO or SAG, then immunostained for Smo (red), Tub^Ac^ (blue), and γ-tubulin (green). (D) Quantitation of Smo ciliary intensity in DMSO or SAG treated limb bud fibroblasts. (E) Limb bud fibroblasts treated with DMSO or SAG, then immunostained for Gpr161 (red), Tub^Ac^ (blue), and γ-tubulin (green). (F) Quantitation of Gpr161 ciliary intensity in DMSO or SAG treated limb bud fibroblasts. Error bars represent standard error of the mean. Statistical significance according to unpaired Student’s *t*-tests (* *p*<0.05). Scale bars, 2 μm.

As cilia are required for vertebrate Hh signaling, we examined whether *Tctn1* ^-/-^ and *Tctn1* ^-/-^ *Nphp4* ^n/n^ mutant cells could respond to Hh pathway stimulation. Upon stimulation with Smo agonist (SAG), a Hh pathway agonist [60, 61], control and *Nphp4* ^n/n^ limb bud fibroblasts induced Hh target genes *Gli1* (MIM 165220) and *Ptch1* (MIM 601309) (Fig 7A and 7B). In contrast, *Tctn1* ^-/-^ *and Tctn1* ^-/-^ *Nphp4* ^n/n^ mutants did not increase *Gli1* and *Ptch1* transcription in response to SAG, indicating defective Hh signal transduction (Fig 7A and 7B).

To further assess the role of Tctn1 and Nphp4 in Hh signaling, we examined the localization of Hh pathway components in those mutant cells that were ciliated. Upon SAG treatment, Smo translocated into the cilia of control and *Nphp4* ^n/n^ cells, but failed to do so in *Tctn1* ^-/-^ and *Tctn1* ^-/-^ *Nphp4* ^n/n^ cells (Fig 7C and 7D). *Tctn1* ^-/-^ and *Tctn1* ^-/-^ *Nphp4* ^n/n^ cilia also failed to localize Gpr161 (Fig 7E and 7F), a negative regulator of the Hh pathway that exits cilia upon pathway activation [62]. *Tctn1* ^-/-^ and *Tctn1* ^-/-^ *Nphp4* ^n/n^ cells therefore have defects in Hh signal transduction, and disrupted ciliary localization of some Hh pathway components.

### Tctn1 and Bbs1 have overlapping functions in mammalian ciliogenesis

As the genetic interactions between MKS and NPHP complex genes in *C*. *elegans* were conserved in mouse, we examined genetic interactions between mouse MKS and NPHP complex genes and BBS-associated genes to determine if these genetic interactions were also conserved. As described above, we observed a synthetic interaction between *C*. *elegans* NPHP complex genes and *bbs-5* (Fig 4A and 4B). Because a *Bbs5* mouse model has not been described, we investigated the function of another BBS-associated gene, *Bbs1* (MIM 209901). *Bbs1* ^-/-^ mutant mice are obese, lack sperm flagella, and develop retinal degeneration, but do not exhibit either the polydactyly or renal abnormalities observed in human BBS-affected individuals [63]. Mouse *Nphp4* ^n/n^ *Bbs1* ^-/-^ double mutant embryos survived through the end of embryogenesis with no gross abnormalities, similar to each single mutant (S4B Fig), indicating that, in contrast to the genetic interaction between *nphp-4* and a BBS-associated gene in *C*. *elegans*, there is no strong genetic interaction between mouse *Nphp4* and *Bbs1*.

**Fig 8:**
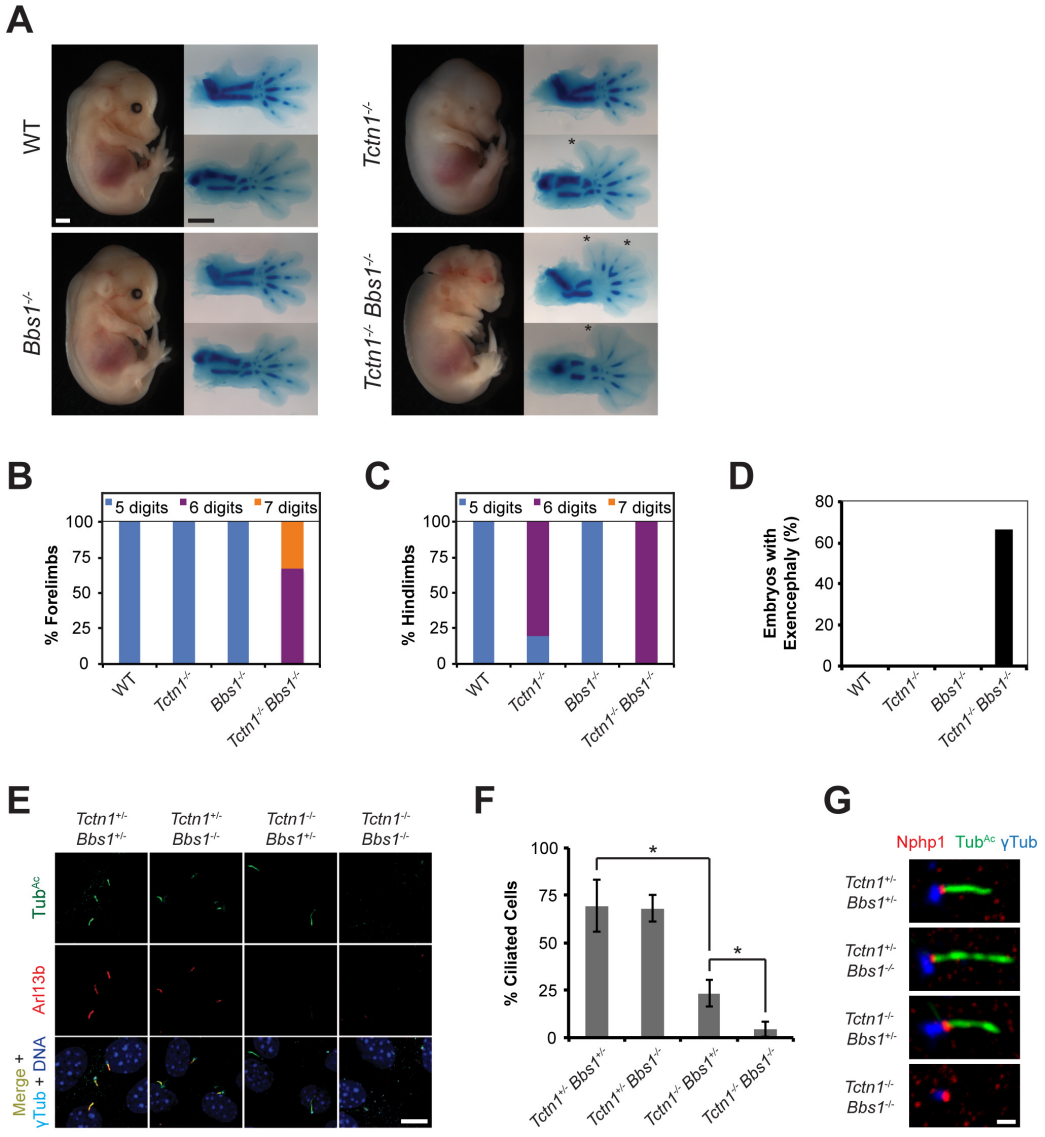
Mouse *Tctn1* genetically interacts with *Bbs1*. (A) Lateral views of wild type, *Tctn1* ^-/-^, *Bbs1* ^-/-^ and *Tctn1* ^-/-^ *Bbs1* ^-/-^ embryos at E14.5. Exencephaly is apparent in the *Tctn1* ^-/-^ *Bbs1* ^-/-^ double mutant. Alcian blue staining of the corresponding genotype’s forelimb (top) and hindlimb (bottom) are included, with asterisks indicating extra digits. Scale bars, 1 mm. (B-D) Number of digits in the (B) forelimbs and (C) hindlimbs, and (D) the incidence of exencephaly for the indicated genotypes. Numbers of animals analyzed for polydactyly and exencephaly are shown in S3 and S4 Tables. (E) Limb bud fibroblasts of the indicated genotypes immunostained for Tub^Ac^ (green), Arl13b (red), γ-tubulin (cyan), and DAPI (blue). Scale bar, 10 μm. (F) Quantification of proportion of ciliated limb bud fibroblasts. Error bars represent the standard deviation. Statistical significance according to unpaired Student’s *t*-tests (* *p*<0.0001). (G) Limb bud fibroblasts immunostained for Nphp1 (red), Tub^Ac^ (green), and γ-tubulin (blue). Scale bar, 1 μm.

Unlike *C*. *elegans*, in which *tctn-1* and *bbs-5* did not interact, mouse *Tctn1* and *Bbs1* did genetically interact. *Tctn1* ^-/-^ *Bbs1* ^-/-^ double mutant embryos displayed dramatically increased polydactyly and exencephaly compared to either single mutant embryos (Fig 8A–8D, S3 and S4 Tables). Thus, a BBS-associated gene interacts with NPHP complex genes in *C*. *elegans*, but not in mouse, whereas a BBS-associated gene interacts with an MKS complex gene in mouse, but not in *C*. *elegans*. BBSome and transition zone complex genes interact in both organisms, but the specific genetic interactions differ between nematodes and mammals, suggesting that some ciliopathy gene interactions are species and/or cell-type specific, and may reflect evolutionary differences in the function of the nematode and mammalian MKS and NPHP complexes.

To analyze the cell biological basis of the genetic interaction between *Tctn1* and *Bbs1*, we examined the cilia of fibroblasts derived from developing forelimb buds. *Tctn1* ^-/-^ *Bbs1* ^-/-^ double mutant cells failed to form cilia, as detected by immunostaining for acetylated tubulin and Arl13b (Fig 8E and 8F). However, *Tctn1* ^-/-^ *Bbs1* ^-/-^ cells still possessed basal bodies, as identified by γ-tubulin staining (Fig 8E). Even when lacking axonemes, *Tctn1* ^-/-^ *Bbs1* ^-/-^ cells still localized Nphp1 appropriately to the transition zone (Fig 8G), unlike *Nphp4* ^n/n^ or *Tctn1* ^-/-^ *Nphp4* ^n/n^ cells (Fig 6E), suggesting that Tctn1 and Bbs1 have overlapping roles in mammalian ciliogenesis, but not in basal body or transition zone formation.

**Fig 9:**
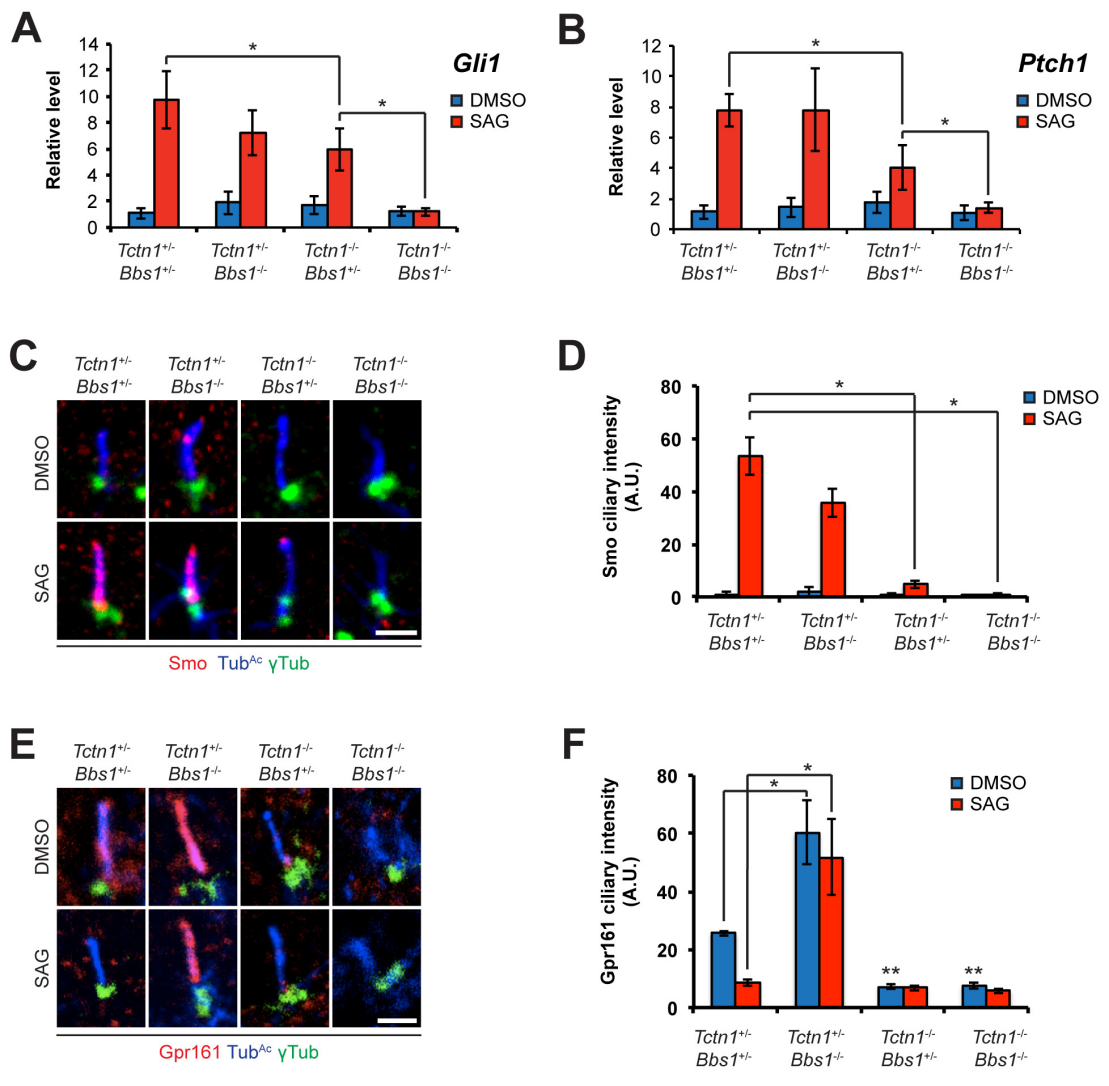
Mouse *Tctn1* and *Bbs1* have overlapping roles in Hh signaling and ciliary localization of Hh pathway components. (A and B) mRNA levels of *Gli1* and *Ptch1* normalized to *β-actin* in forelimb bud cells treated with DMSO or SAG. Error bars represent the standard deviations. Statistical significance according to unpaired Student’s *t*-tests (* *p*<0.05). (C) Limb bud fibroblasts treated with DMSO or SAG, then immunostained for Smo (red), Tub^Ac^ (blue), and γ-tubulin (green). (D) Quantitation of Smo ciliary intensity in DMSO or SAG treated limb bud fibroblasts. *p<0.05 in unpaired Student’s *t*-tests. (E) Limb bud fibroblasts treated with DMSO or SAG, then immunostained for Gpr161 (red), Tub^Ac^ (blue), and γ-tubulin (green). (F) Quantitation of Gpr161 ciliary intensity in DMSO or SAG treated limb bud fibroblasts. *p<0.01 and **p<0.001 in unpaired Student’s *t*-tests with respect to *Tctn1* ^+/-^ *Bbs1* ^+/-^ mutant fibroblasts treated with DMSO. Error bars represent standard error of the mean. Scale bars, 2 μm.

We hypothesized that the dramatic loss of cilia in *Tctn1* ^-/-^ *Bbs1* ^-/-^ double mutants compared to *Tctn1* ^-/-^ single mutants could account for the increased polydactyly of *Tctn1* ^-/-^ *Bbs1* ^-/-^ embryos, as decreased ciliary function and consequent decreased Gli3 processing results in polydactyly [10]. To test this possibility, we measured the mRNA expression levels of the Shh target genes *Gli1* and *Ptch1* in limb bud fibroblasts stimulated with SAG. *Tctn1* ^-/-^ mutant fibroblasts showed a reduction in *Gli1* and *Ptch1* expression compared to controls, and *Tctn1* ^-/-^ *Bbs1* ^-/-^ double mutants showed a further reduction compared to *Tctn1* ^-/-^ mutants (Fig 9A and 9B). Thus *Tctn1* ^-/-^ *Bbs1* ^-/-^ double mutants have abrogated Hh pathway activation, likely resulting in the exacerbation of ciliopathy phenotypes compared to either *Tctn1* or *Bbs1* single mutants.

To further probe the role of Tctn1 and Bbs1 in Hh signal transduction, we examined the localization of the Hh pathway components Smo and Gpr161. Consistent with reduced Shh signaling, and in contrast to control and *Bbs1* ^-/-^ fibroblasts, Smo failed to translocate to cilia upon SAG treatment in *Tctn1* ^-/-^ and *Tctn1* ^-/-^ *Bbs1* ^-/-^ fibroblasts (Fig 9C and 9D). Gpr161, a negative regulator of Hh signaling, was present in cilia of control cells and was removed from cilia upon SAG treatment. Unexpectedly, *Bbs1* ^-/-^ mutant cells displayed increased levels of Gpr161 in cilia. Moreover, Gpr161 remained in *Bbs1* ^-/-^ mutant cilia upon SAG treatment, demonstrating that Bbs1 is required for the removal of Gpr161 from cilia (Fig 9E and 9F). *Tctn1* ^-/-^ cilia lacked Gpr161 even in the absence of SAG, as did *Tctn1* ^-/-^ *Bbs1* ^-/-^ double mutant cilia (Fig 9E and 9F). Thus, *Bbs1* promotes the exit of Gpr161 from cilia and *Tctn1* promotes the localization of Gpr161 to cilia.

## Discussion

**Fig 10:**
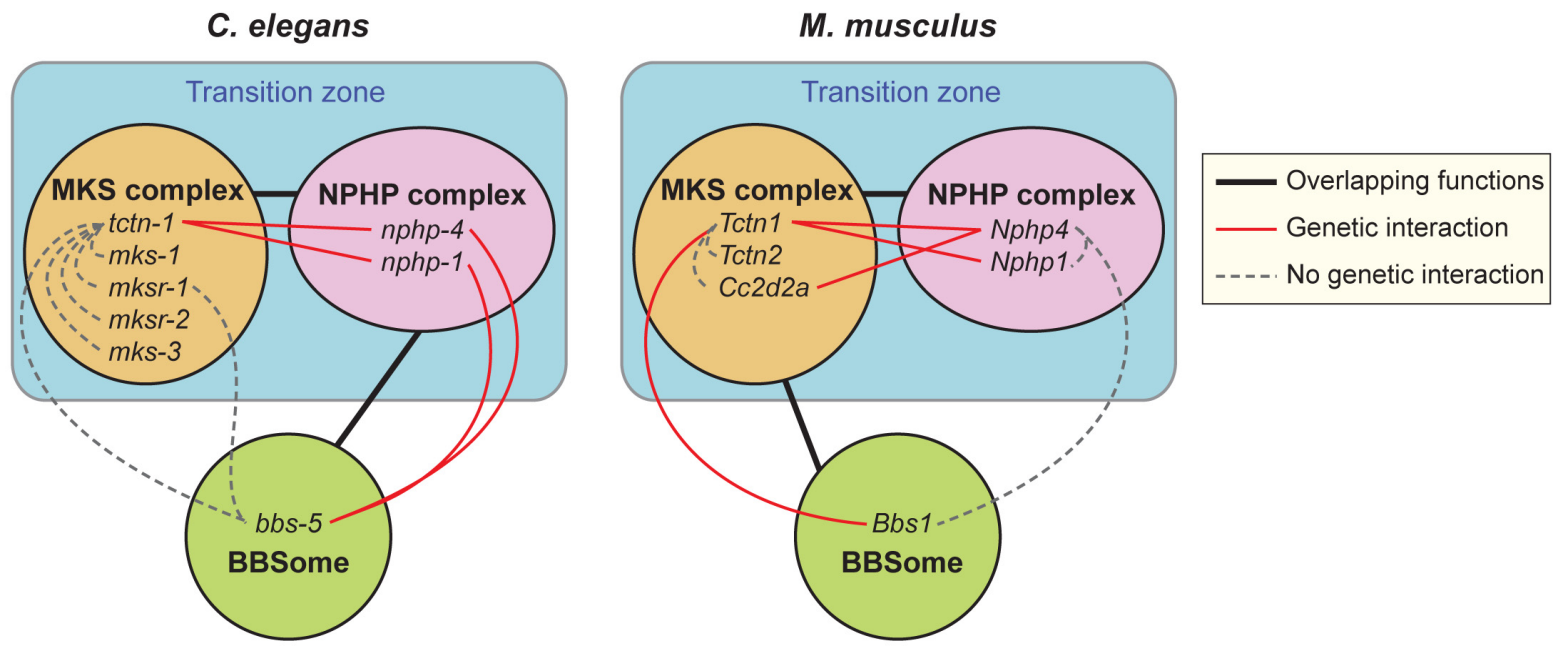
Genetic interactions between transition zone complexes and the BBSome in *C*. *elegans* and mice. In *C*. *elegans*, MKS complex genes interact with NPHP complex genes, and a BBS-associated gene interacts with NPHP complex genes. In *M*. *musculus*, MKS complex genes interact with NPHP complex genes, and a BBS-associated gene interacts with at least one MKS complex gene. Tctn1 behaves as an MKS component in both organisms. Protein complexes that share overlapping functions in ciliogenesis are connected by black lines. Within protein complexes, specific genes that display a genetic interaction are connected by red lines. Genes that show no genetic interaction are connected by dashed grey lines.

We investigated the genetic interactions between distinct biochemical complexes involved in ciliary function in both nematodes and mammals. We found that TCTN-1, the *C*. *elegans* ortholog of the mammalian Tectonics, localized to the ciliary transition zone. Loss of *tctn-1* did not abrogate ciliogenesis on its own or in combination with mutations affecting other MKS complex components. However, loss of *tctn-1* in combination with loss of components of the NPHP complex, a biochemically distinct transition zone complex, synergistically abrogated ciliogenesis (Fig 10). This interaction between MKS and NPHP complex genes also occurred in mice, suggesting that the overlapping functions of the MKS and NPHP complexes in supporting ciliary structure are evolutionarily conserved. To extend our understanding of how different ciliopathy protein complexes work together, we analyzed how BBS-associated proteins function with transition zone complexes. Components of the BBSome cooperate with MKS and NPHP complexes, but the specific genetic interactions that support ciliogenesis in *C*. *elegans* and mice differ (Fig 10). Thus, we have uncovered evolutionarily conserved and non-conserved functional interactions between three biochemically distinct ciliopathy complexes.

In both mammals and *C*. *elegans*, MKS and NPHP complex proteins localize to the transition zone at the ciliary base. Both the mouse and *C*. *elegans* orthologs of Tctn1 possess signal peptides, and are thus predicted to localize to the extracellular side of the transition zone. Where Tctn1 is relative to the remainder of the MKS complex, the NPHP complex, and the electron-micrographically defined components of the transition zone, such as the Y-links, remains to be determined. How the transition zone regulates the protein composition of cilia is not well understood, though the Y-links themselves have been implicated [13, 14, 40]. We found that *C*. *elegans nphp-4* and *tctn-1; nphp-4* mutant cilia had missing or reduced Y-links, but still localized ARL-13 and ODR-10, suggesting that the presence of intact Y-links is not critical for all ciliary protein localization. Additional analyses will be required to clarify the composition of Y-links and the relationship of Y-link structure to the control of ciliary composition.

Strikingly, Tctn1 was required for the ciliary localization of Arl13b in mouse, but not for the ciliary entry of ARL-13 in *C*. *elegans*, a difference that suggests that Tctn1 or the transition zone function distinctly in mammals and nematodes. In addition to Arl13b, which associates with membranes through palmitoyl anchors [42], mouse Tctn1 is required for the ciliary localization of other membrane-associated proteins, such as Smo, Pkd2 and Inpp5e [16, 26, 64]. We found that *C*. *elegans* TCTN-1 controls ciliary membrane composition similarly to other transition zone proteins, by excluding the non-ciliary membrane protein TRAM-1a from the cilium [13]. One possibility is that the mammalian MKS complex functions primarily to promote the localization of ciliary membrane-associated proteins, whereas the nematode MKS complex functions primarily to prevent the entry of non-ciliary membrane-associated proteins.

We found a genetic interaction between transition zone genes and BBS-associated genes. Intriguingly, the NPHP complex synergizes with a BBS-associated gene in *C*. *elegans* whereas an MKS complex gene synergizes with a BBS-associated gene in mice (Fig 10). No interactions were found between the MKS complex genes and a BBS-associated gene in *C*. *elegans* or between an NPHP complex gene and a BBS-associated gene in mice. Functional differences between *bbs-5* and *Bbs1* may account for the different genetic interactions observed in nematodes and mice. Despite these limitations, the specificity of the synergistic interactions between *Bbs1* and *Tctn1* in mice, and between *bbs-5* and *nphp-4* or *nphp-1* in *C*. *elegans* suggests that the transition zone has altered its relationship to the BBSome through evolution. Intriguingly, the NPHP complex in *C*. *elegans* also localizes to the basal body-transition fibers, where the BBSome and IFT proteins dock [13, 40]. It will be interesting to determine whether the precise locations or functions of the MKS and NPHP complexes in the transition zone are different in nematodes and mammals, and whether these differences account for the distinct interactions with the BBSome.

How compromising the MKS and NPHP complexes, MKS and BBS complexes in mammals, or NPHP and BBS complexes in nematodes results in synthetic ciliary defects remains unclear. In mammals, Tctn1 is required for the localization of select transition zone proteins and a subset of ciliary membrane proteins [16]. Given that the BBSome facilitates the ciliary localization of other membrane proteins [29], compromising both the BBSome and MKS complex function may abrogate transport sufficiently to disrupt ciliary function and ciliogenesis. Physical interaction between Tctn1 and the BBSome has not been detected, but intriguingly, the MKS complex interactor, Cep290, binds to Bbs4 [16, 32]. Furthermore, loss of Bbs4 exacerbates the mislocalization of Rhodopsin in *Cep290* mutant retinas [32], indicating that MKS complex interactors and the BBSome can cooperate to transport proteins to cilia.

BBS-associated genes have also been implicated in the export of proteins from cilia [65–68]. Our finding that Bbs1 is required for the ciliary exclusion of Gpr161, whereas Tctn1 is required for the ciliary localization of Gpr161, suggests that the BBSome and the transition zone may also act antagonistically to dynamically localize some ciliary proteins. Arl6, a BBSome-associated GTPase, is similarly required to remove Gpr161 from cilia [69]. This shared requirement suggests that removing Gpr161 from cilia may involve most or all BBS-associated proteins.

As the MKS and NPHP complexes also have overlapping functions in supporting ciliogenesis in mice, the NPHP complex may, like the MKS complex, regulate protein trafficking to cilia. Likewise, in *C*. *elegans*, in which the NPHP complex genes interact with both MKS and BBS genes, the NPHP complex may have overlapping functions in regulating ciliary protein composition. In support of this possibility, *C*. *elegans* NPHP-4 is required for proper ciliary localization of certain IFT proteins and *Chlamydomonas* Nphp4 regulates the entry of a subset of membrane proteins into flagella [25, 70].

As phenotypes reflect gene function, new phenotypes generated by the interaction of multiple mutations can reveal previously unknown functions of the genes involved. On their own, *Nphp4* and *Nphp1* do not play critical roles in limb bud or neural tube development. However, mutation of *Nphp4* or *Nphp1* modifies the *Tctn1* ^-/-^ limb and neural tube phenotypes, thereby demonstrating their involvement in the affected tissues. Similarly, human BBS patients have extra digits, yet mouse models of BBS have thus far not displayed polydactyly. Our results demonstrate that mouse *Bbs1* does indeed affect limb patterning, exposed in the context of a *Tctn1* ^-/-^ mutation. Therefore, studying genes in combination can reveal roles for those genes that might otherwise be masked by the overlapping function of other genes. By identifying overlapping functions for genes in model organisms, particularly those from distinct biochemical complexes, we may be able to predict which genes interact and which do not in humans.

Thus far, understanding how human genotypes predict phenotypes is insufficient except for the most straightforward traits. For ciliopathy-affected individuals, even when the principal disease-causing mutation is identified, how mutations in that gene manifest as a range of phenotypes in different populations remains unknown. Genome-wide association studies (GWAS) have identified polymorphisms associated with many diseases and traits. However, these only account for a small percentage of the estimated heritability, provoking searches for sources of “missing heritability” [71, 72]. Work in simple organisms such as yeast has suggested that more extensive accounting for the effects of allelic variation can explain the phenotypic variation in disease and common traits [73]. Our work suggests that in heterogeneous, oligogenic disorders such as ciliopathies, missing heritability could arise not from the effects of polymorphisms by themselves but from gene specific interactions between polymorphisms, which GWAS fail to detect. Our double mutant analyses demonstrate that the phenotypes that arise for a given mutation depend on the genetic context, such that an innocuous mutation in one genetic background may alter a disease phenotype in another. In support of this possibility in the specific case of ciliopathies, human genetic studies have found NPHP and BBS patients with multiple genetic lesions that contribute to their phenotypes [9, 74, 75]. As common or rare variants may be shared within a family, they may be a source of heritability and significant modifiers of disease phenotypes. Detecting variants that affect expressivity only in specific genetic contexts will require power and computation not currently present in GWAS. Therefore, as a complement to GWAS, animal genetic models provide a valuable means to identify the oliogenic interactions that define inherited disease phenotypes.

## Materials and Methods

### Ethics statement

Mouse protocols were approved by the Institutional Animal Care and Use Committee at the University of California, San Francisco (approval AN091380). Euthanasia was performed by cervical dislocation, and all animal studies were conducted in accordance with internationally-accepted standards.

### *C*. *elegans* strains

All strains (S5 Table) were generated and maintained under standard conditions [76]. The *tctn-1* mutant strain, E04A4.6*(ok3021)* IV (WormBase ID: WBVar00094107), was obtained from the *C*. *elegans* Gene Knockout Consortium via the *Caenorhabditis* Genetics Center and outcrossed to wild type (N2) six times. Standard mating procedures were used to generate double mutants and to introduce fluorescently tagged protein constructs into the specified genetic backgrounds.

### Subcellular protein localization

Strains bearing TCTN-1 C-terminal GFP translational fusion constructs were generated by stitch-PCR, essentially as previously described [77]. In brief, the *bbs-8* promoter region, followed by the complete genomic coding region of the *tctn-1* gene, was fused in-frame to EGFP, and used to create transgenic lines. Live animals were anaesthetized using 10 mM levamisole, mounted on 5% agarose pads, and observed by spinning-disc confocal microscopy. The subcellular localization patterns of the fluorescent marker-tagged proteins were assessed in either wild type (N2) animals or in the indicated mutant backgrounds. Mislocalization phenotypes were confirmed by analyzing at least 50 animals for each strain. For visualization of ODR-10::GFP, animals were fixed and immunostained with anti-GFP (600-101-215; Rockland) following the Finney-Ruvkun protocol [78], and imaged on a Leica TCS SPE confocal microscope.

### *C*. *elegans* assays

To assess sensory neuron dye filling, 300 synchronized L4 worms were washed with S-basal and immersed in 10 mg/ml DiI in S-basal for two hours. Worms were rinsed with S-basal, and plated onto an OP50 seeded plate. After one hour, the animals were anesthetized with 0.2 M sodium azide, mounted on a 2% agarose pad, and imaged on a Zeiss Axioplan microscope. Assays were performed on at least three separate occasions for each genotype. Statistical significance was assessed using the unpaired Student’s *t* test.

To measure growth, 150 synchronized L1 larvae were plated on OP50 and incubated at 20°C. After 45 hours, the animals were counted and staged. Assay was repeated three times. To measure size, DIC images of synchronized day 1 gravid adults were captured on a Zeiss Axioplan microscope. Perimeter and area were measured using Fiji software for 20–35 animals of each genotype. Egg laying was measured by picking synchronized day 1 gravid adults onto individual plates. One hour later, the number of eggs on the plate was counted. Brood size was measured by plating synchronized L4 animals, and transferring the animals onto fresh plates daily until the end of the reproductive period. Progeny from each plate was counted for at least ten animals. Standard osmotic avoidance and chemotaxis assays were performed as previously described using 8M glycerol to create high osmolarity, and diacytyl (1:1000 dilution) and butanone (1:1000 dilution) as chemoattractants [79, 80].

### Mouse alleles and mutant analysis

*Tctn1* ^−^(*Tctn1* ^*Gt(KST296)Byg*^), *Tctn2* ^−^(*Tctn2* ^*tm1*.*1Reit*^), *Nphp4* ^n^(*Nphp4* ^*nmf192*^), *Nphp1* ^−^(*Nphp1* ^*tm1Jgg*^) *Cc2d2a* ^−^(*Cc2d2a* ^*Gt(AA0274)Wtsi*^), and *Bbs1* ^−^(*Bbs1* ^*tm1Vcs*^) alleles have been previously described [16, 21, 33, 50, 63, 81]. Single and double mutant embryos were generated by timed matings of trans-heterozygous animals. For gross phenotypic examination, embryos were harvested at E14.5, fixed in 4% paraformaldehyde, and imaged on a Zeiss Discovery V12 steREO microscope. At least three embryos of each double mutant combination were analyzed. Limb bud digits were visualized by fixing E14.5 embryos in ethanol and staining in Alcian blue and Alizarin red, as previously described [82].

### Limb fibroblast derivation

Limb fibroblast cell lines were generated from E13.5 mouse embryos that were harvested and transferred into Dulbecco's PBS with penicillin, streptomycin and fungizone (P/S/F) at 37°C. For each embryo, both forelimb buds were extracted and incubated for 2 minutes at room temperature in 0.05% Trypsin-EDTA solution, after which they were disaggregated by pipetting. The resulting cell suspensions were immediately transferred into DMEM medium supplemented with 20% fetal bovine serum (FBS) and P/S/F, and incubated overnight with gentle shaking at 37°C and 5% CO_2_. The next day cells were confluent and were passaged using the trypsinization procedure described above. Cells were maintained in DMEM+15%FBS+P/S/F and were not diluted more than five times when passaging. All analyses were performed in cells passaged less than ten times.

### Immunofluorescence

For immunostaining, confluent limb bud fibroblasts were starved for 48 hours in OptiMEM medium to induce ciliation. For immunostaining of Smo or Gpr161, confluent limb bud fibroblasts were starved for 24 hours in Optimem, and treated DMSO or SAG (1 μM) for 6 hours. Cells were fixed by incubating them first in DPBS+4% paraformaldehyde (10 min, RT), followed by cold methanol (3 min, -20°C). Cells were blocked and permeabilized for at least 15 min at RT in blocking solution (PBS+2% donkey serum+0.1% Triton-X100+0.02% sodium azide). Coverslips were then incubated with primary antibodies diluted in blocking solution for 3 hours at RT. After two rinses in PBS, coverslips were incubated with fluorophore (Alexa Fluor)-conjugated donkey secondary antibodies and DAPI for 1 hour at RT in darkness. This was followed by two rinses in milliQ water and the mounting of coverslips on slides using gelvatol mounting medium. Slides were imaged on a Leica TCS SPE confocal microscope. Antibodies used were: acetylated tubulin (clone 6-11B-1; Sigma), γ-tubulin (C-20; Santa Cruz), Arl13b (17711-1-AP; Proteintech), Nphp1 (gift from Dr. Greg Pazour), Smoothened (ab38686; Abcam) and Gpr161 (13398-1-AP; Proteintech). For quantitation of cilia numbers, fields of cells were imaged at seven positions spanning the whole sample. Acetylated tubulin-positive cilia associated with a γ-tubulin-positive basal body were counted in each field and normalized to the number of nuclei in the field. Statistical significance was assessed using the unpaired Student’s *t* test. Quantification of Smo and Gpr161 ciliary intensity and immunostaining of limb bud cryosections were performed as previously described [16, 64].

### Shh signaling assay

To measure Shh signaling, confluent limb bud fibroblasts were starved in OptiMEM for 24 hours, then treated with DMSO or 200nM SAG (Cayman Chemical) for 24 hours. RNA was extracted from these cells using the RNeasy Mini Kit (Qiagen) and cDNA was synthesized using the SuperScript III First-Strand Synthesis System (Invitrogen). Quantitative PCR was performed using EXPRESS SYBR GreenER qPCR Supermix, with premixed ROX (Invitrogen) on a 7300 Real-Time PCR machine (Applied Biosystems). Transcript levels of *Gli1* and *Ptch1* were normalized to levels of *β-actin*. The primers used were Gli1F, 5’-GGTGCTGCCTATAGCCAGTGTCCTC-3’; Gli1R, 5’-GTGCCAATCCGGTGGAGTCAGACCC-3’; Ptch1F, 5’-CTCTGGAGCAGATTTCCAAGG-3’; Ptch1R, 5’-TGCCGCAGTTCTTTTGAATG-3’; β-actinF, 5’-CACAGCTTCTTTGCAGCTCCTT-3’; and β-actinR, 5’-CGTCATCCATGGCGAACTG-3’. Experiments were performed in triplicate and repeated at least three times. Statistical significance was assessed using the unpaired Student’s *t* test.

### Electron microscopy

Transmission electron microscopy of *C*. *elegans* amphid cilia was performed as previously described [13, 42]. Images depicting *nphp-4* single mutant cilia were of strain PT709: *nphp-4(tm925) him-5(e1490)* V. No differences in ciliary structure were detected between *nphp-4* and *nphp-4 him-5* genotypes (Lambacher *et al*., manuscript in revision). For TEM analysis of limb cilia in mice, we isolated forelimb buds from E11.5 embryos, washed with Dulbecco's PBS and incubated in fixative solution containing 0.1M sodium cacodylate (pH 7.2), 4% formaldehyde, 4% glutaraldehyde, 4% tannic acid and 5% sucrose, where they remained for two days. The limb buds were processed for TEM as previously described [16]. Images were acquired using a JEOL JEM-1400 electron microscope. For quantification of limb bud cilia, axonemes with docked basal bodies were counted per field in at least seven fields. Statistical significance was assessed using the unpaired Student’s *t* test.

## Supporting Information

S1 Fig Orthologs of Tectonic1. Sequence alignment of Tectonic1 orthologs of *Homo sapiens*, *Mus musculus*, *Ciona intestinalis* (sea squirt), *Drosophila virilis* (fruit fly), *Aedes aegypti* (mosquito), *Nematostella vectensis* (sea anemone), *Trichoplax* (Placozoan), *Monosiga brevicollis* (choanoflagellate), *Naegleria gruberi* (amoeboflagellate), *Batrachochytrium dendrobatidis* (chytrid fungus), *Tetrahymena thermophile*, *Trypanosome brucei*, *Chlamydomonas reinhardtii*, and *Caenorhabditis elegans*. Predicted signal peptides are indicated in red. *C*. *elegans tctn-1* is most homologous to other Tectonics in the C-terminal region.
(PDF)

S2 Fig *tctn-1* mutants are grossly normal *in C*. *elegans*. (A) Growth at 20°C, (B) size, (C) egg laying, (D) brood size, (E) response to high osmolarity, and (F) chemotaxis abilities to diacytyl and butanone were assessed in *tctn-1* mutants. Additional single and double mutant genotypes were included as indicated.
(PDF)

S3 Fig Affected cilia in *tctn-1; nphp-4* mutants and unaffected cilia in *tctn-1; mks-3* mutants in *C*. *elegans*. *tctn-1; nphp-4* double mutants display morphologically abnormal cilia. (A) *tctn-1; nphp-4* PHA and PHB cilia, visualized with XBX-1::tdTomato, are slightly shorter compared to controls. (B) Measurements of cilia lengths as shown in (A), * p<0.001, ns: no significant difference. (C) *tctn-1; nphp-4* mutants display mis-oriented PHA and PHB cilia visualized with XBX-1::tdTomato and MKS-6::GFP. (D) Percentage of mis-oriented *tctn-1; nphp-4* cilia as shown in (C) compared to controls. (E) *tctn-1; mks-3* mutants do not have defects in ciliary structure. Low and high magnification TEM cross-sections of the distal segment, middle segment, transition zone, and PCMC of amphid cilia of *tctn-1; mks-3* double mutants. Schematics below (lateral and transverse views). Green arrows indicate intact Y-links at the transition zone. Boxed numbers indicate distances (μm) from the distal ciliary tips. Scale bars,100 nm.
(PDF)

S4 Fig Mouse *Nphp4* does not genetically interact with *Nphp1* or *Bbs1*. (A) *Nphp1* ^-/-^ *Nphp4* ^n/n^ double mutants are viable and grow at the same rate as their littermates of indicated genotypes. Two litters are shown with each line representing one animal. Squares represent males and circles represent females. (B) *Nphp4* ^n/n^ *Bbs1* ^-/-^ double mutant embryos at E17.5 resemble wild type, *Nphp4* ^n/n^ and *Bbs1* ^-/-^ single mutant embryos. Scale bar, 1 cm.
(PDF)

S1 Table Polydactyly in MKS and NPHP complex mutants. The number of limbs with the indicated number of digits observed, with the percentage in parentheses.
(PDF)

S2 Table Exencephaly in MKS and NPHP complex mutants. The number of embryos with exencephaly among the total examined, with the percentage in parentheses.
(PDF)

S3 Table Polydactyly in *Tctn1* ^-/-^ *Bbs1* ^-/-^ mutants. The number of limbs with the indicated number of digits observed, with the percentage in parentheses.
(PDF)

S4 Table Exencephaly in *Tctn1* ^-/-^ *Bbs1* ^-/-^ mutants. The number of embryos with exencephaly among the total examined, with the percentage in parentheses.
(PDF)

S5 Table *C*. *elegans* strains used in this study. (PDF)
