# Supplementary material for: Conserved Genetic Interactions between Ciliopathy Complexes Cooperatively Support Ciliogenesis and Ciliary Signaling
Source: PLoS Genet. 2015 Nov 5;11(11):e1005627. doi: 10.1371/journal.pgen.1005627 (PMC4635004; doi:10.1371/journal.pgen.1005627)
Supplement: S1 Fig — Sequence alignment of Tectonic1 orthologs of Homo sapiens, Mus musculus, Ciona intestinalis (sea squirt), Drosophila virilis (fruit fly), Aedes aegypti (mosquito), Nematostella vectensis (sea anemone), Trichoplax (Placozoan), Monosiga brevicollis (choanoflagellate), Naegleria gruberi (amoeboflagellate), Batrachochytrium dendrobatidis (chytrid fungus), Tetrahymena thermophile, Trypanosome brucei, Chlamydomonas reinhardtii, and Caenorhabditis elegans. Predicted signal peptides are indicated in red. C. elegans tctn-1 is most homologous to other Tectonics in the C-terminal region. (PDF) [file pgen.1005627.s001.pdf]

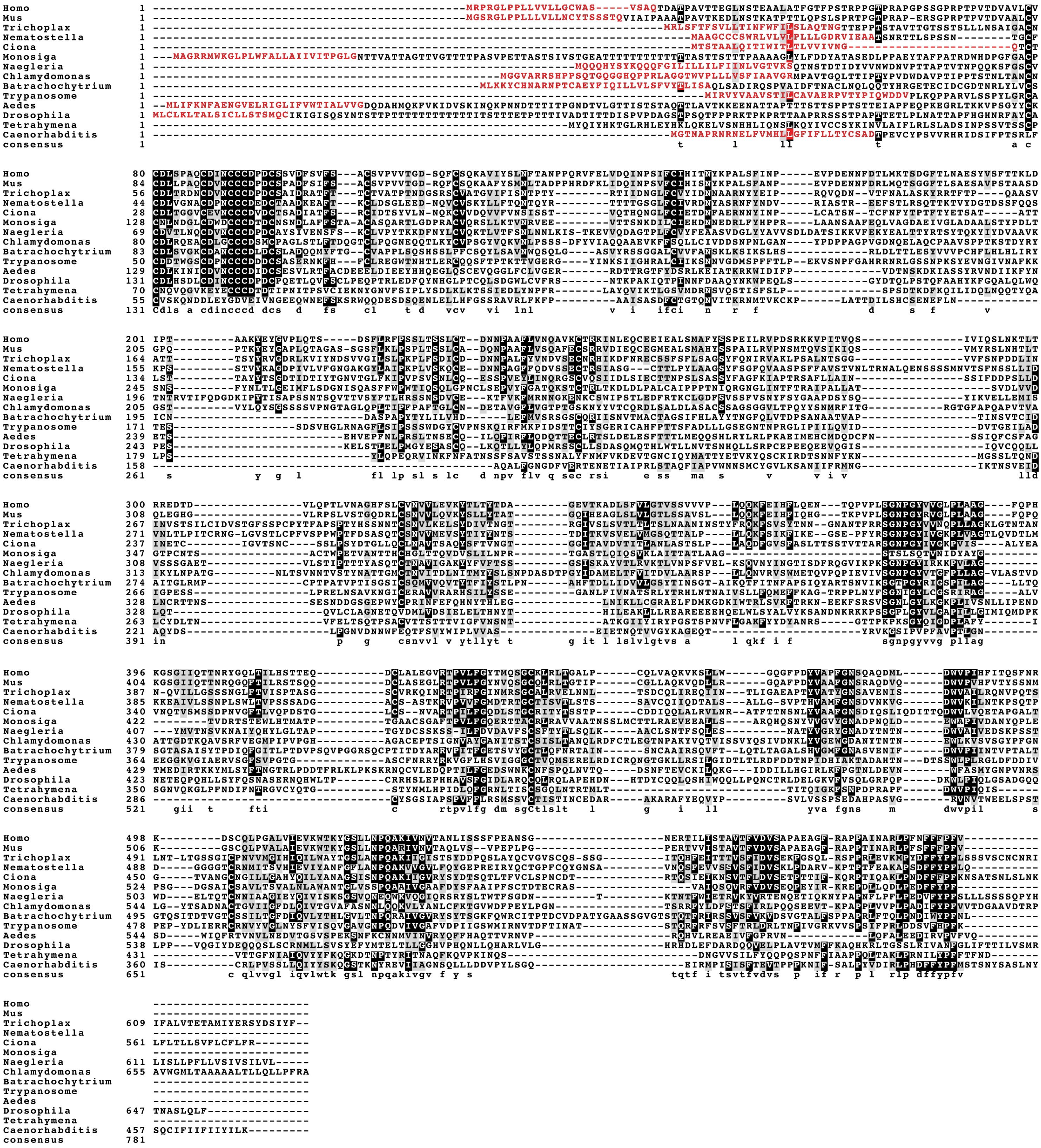
Homo
Mus
Trichoplax
Nematostella
Ciona
Monosiga
Naegleria
Chlamydomonas
Batrachochytrium
Trypanosome
Aedes
Drosophila
Tetrahymena
Caenorhabditis
consensus
