# Supplementary figures and images for: Conserved Genetic Interactions between Ciliopathy Complexes Cooperatively Support Ciliogenesis and Ciliary Signaling
Source: PLoS Genet. 2015 Nov 5;11(11):e1005627. doi: 10.1371/journal.pgen.1005627 (PMC4635004; doi:10.1371/journal.pgen.1005627)

**A**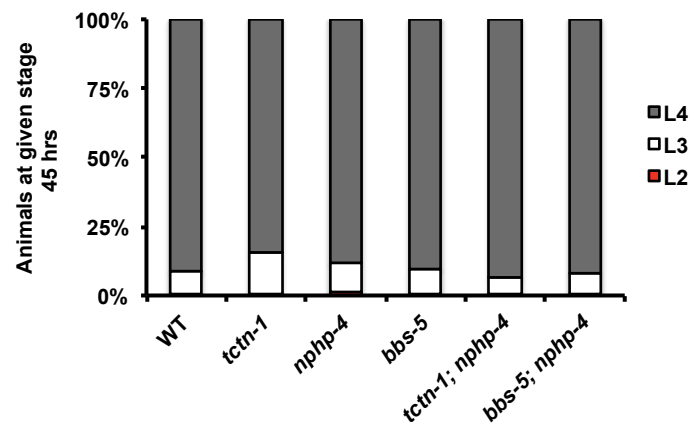**B**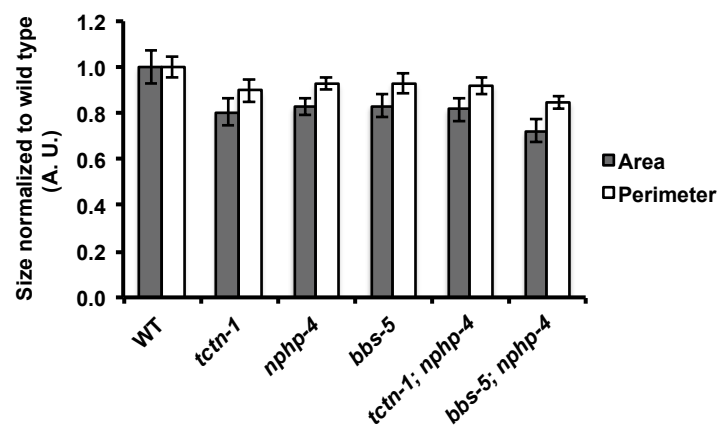**C**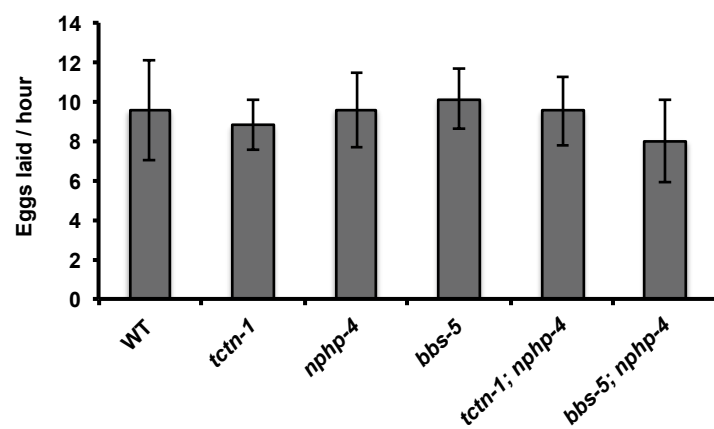**D**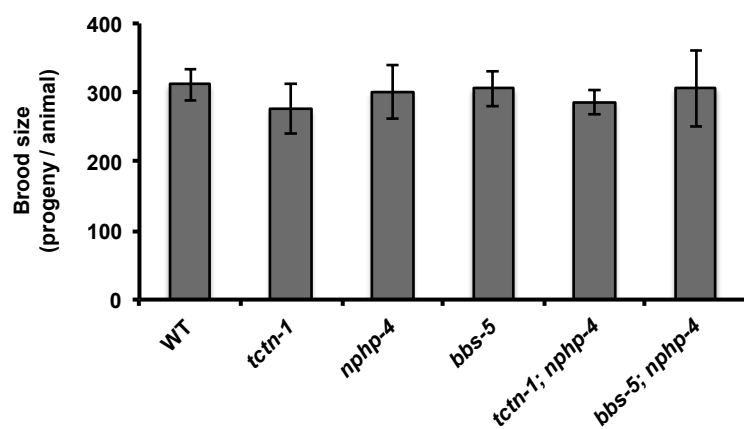**E**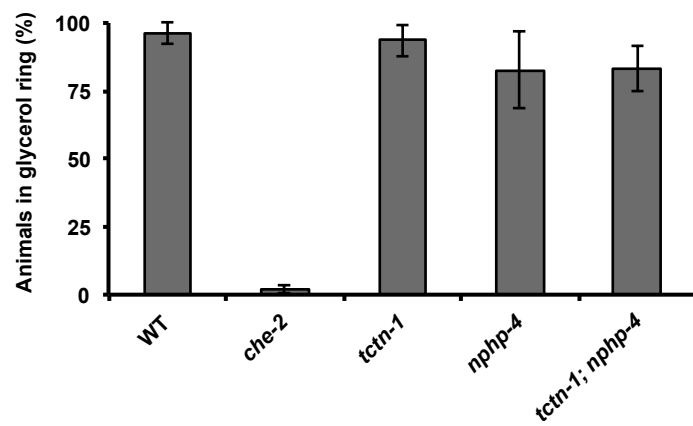**F**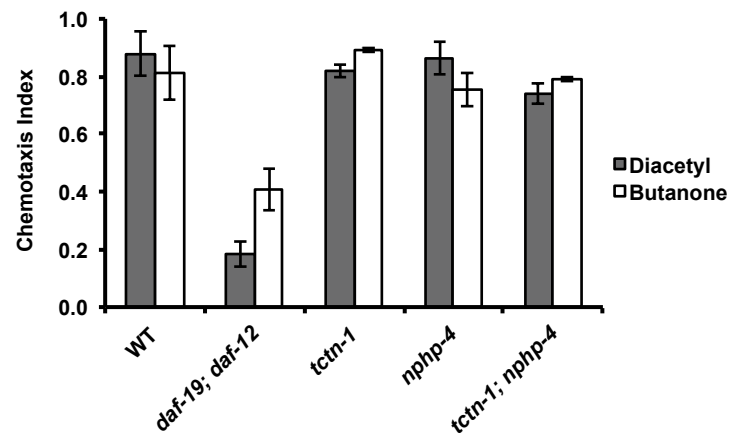

Supplement: S2 Fig — (A) Growth at 20°C, (B) size, (C) egg laying, (D) brood size, (E) response to high osmolarity, and (F) chemotaxis abilities to diacytyl and butanone were assessed in tctn-1 mutants. Additional single and double mutant genotypes were included as indicated. (PDF) [file pgen.1005627.s002.pdf]

**A**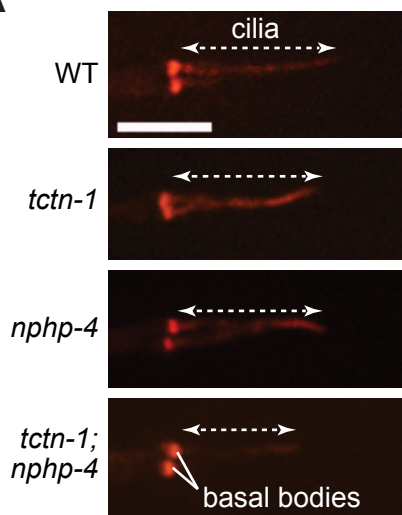**B**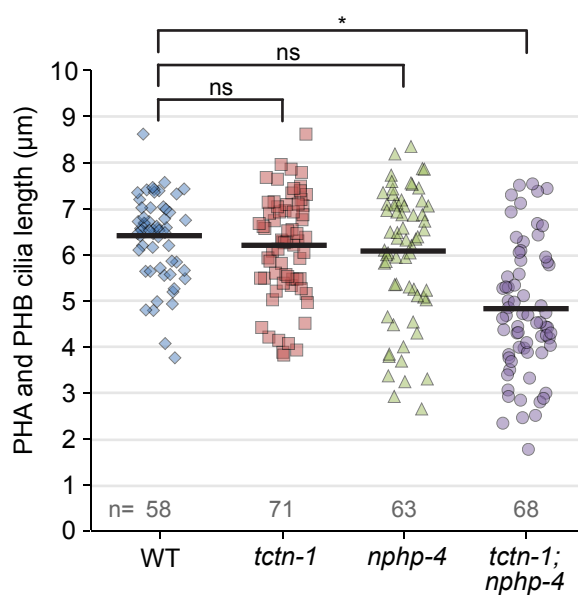**C**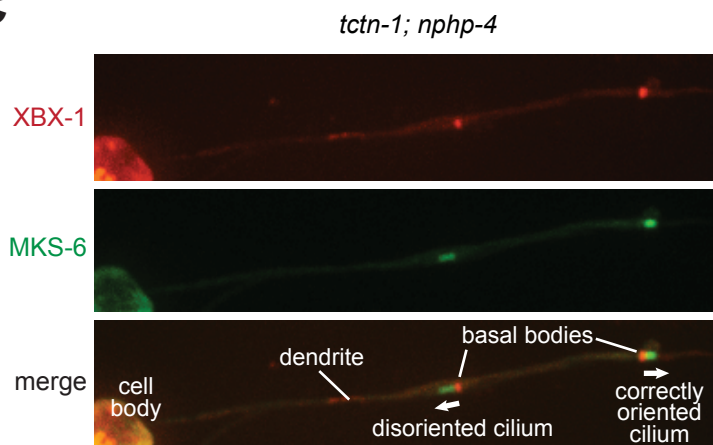**D**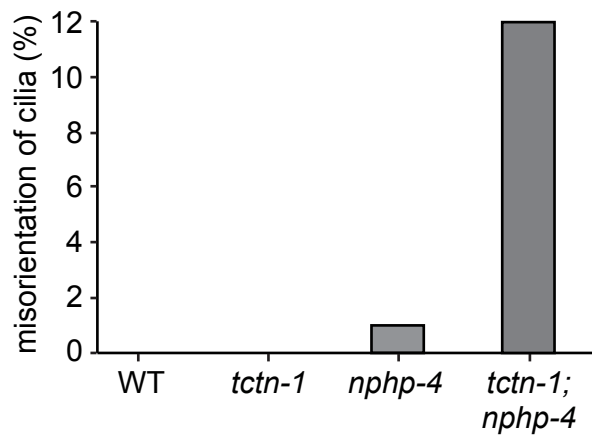**E**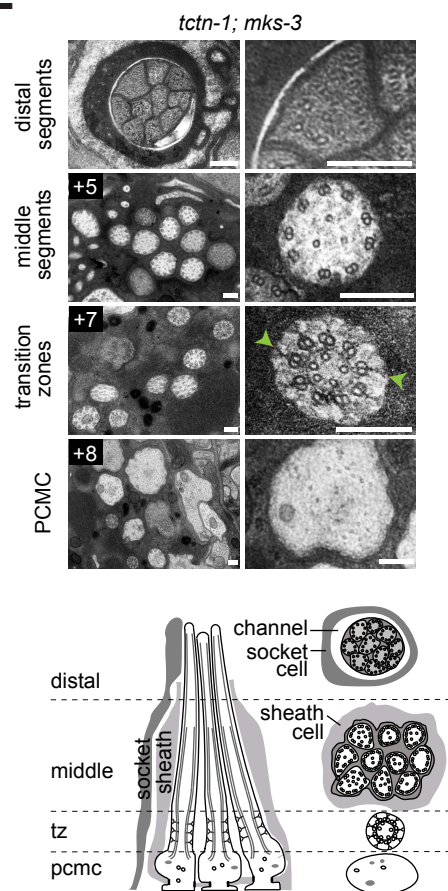

Supplement: S3 Fig — tctn-1; nphp-4 double mutants display morphologically abnormal cilia. (A) tctn-1; nphp-4 PHA and PHB cilia, visualized with XBX-1::tdTomato, are slightly shorter compared to controls. (B) Measurements of cilia lengths as shown in (A), * p<0.001, ns: no significant difference. (C) tctn-1; nphp-4 mutants display mis-oriented PHA and PHB cilia visualized with XBX-1::tdTomato and MKS-6::GFP. (D) Percentage of mis-oriented tctn-1; nphp-4 cilia as shown in (C) compared to controls. (E) tctn-1; mks-3 mutants do not have defects in ciliary structure. Low and high magnification TEM cross-sections of the distal segment, middle segment, transition zone, and PCMC of amphid cilia of tctn-1; mks-3 double mutants. Schematics below (lateral and transverse views). Green arrows indicate intact Y-links at the transition zone. Boxed numbers indicate distances (μm) from the distal ciliary tips. Scale bars,100 nm. (PDF) [file pgen.1005627.s003.pdf]

**A**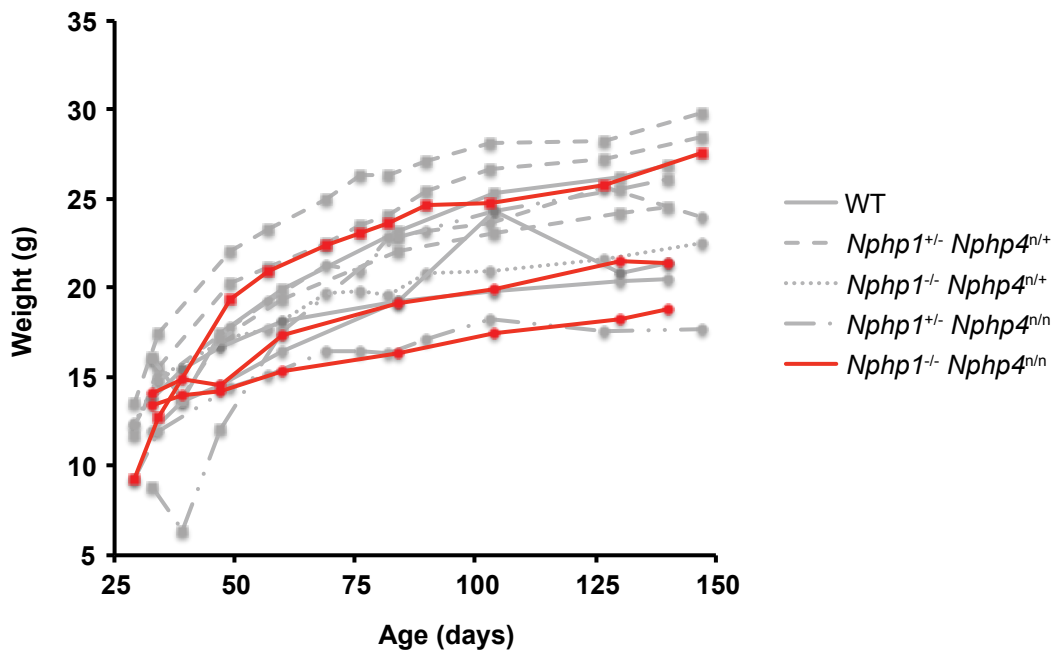**B**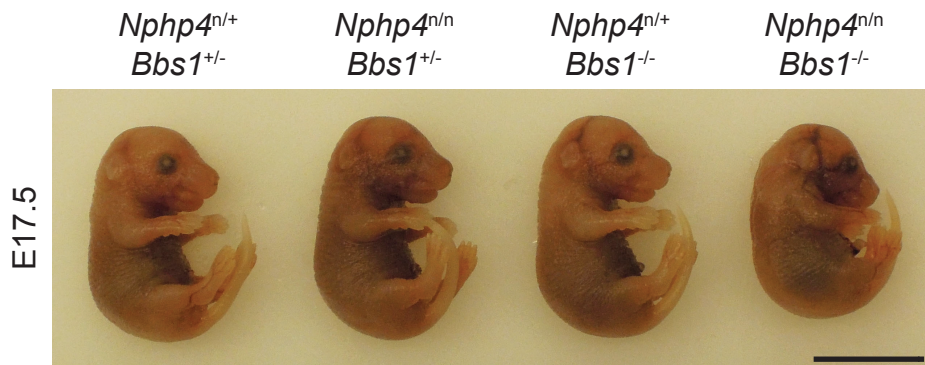

Supplement: S4 Fig — (A) Nphp1 -/- Nphp4 n/n double mutants are viable and grow at the same rate as their littermates of indicated genotypes. Two litters are shown with each line representing one animal. Squares represent males and circles represent females. (B) Nphp4 n/n Bbs1 -/- double mutant embryos at E17.5 resemble wild type, Nphp4 n/n and Bbs1 -/- single mutant embryos. Scale bar, 1 cm. (PDF) [file pgen.1005627.s004.pdf]
