# Supplementary material for: Conserved Genetic Interactions between Ciliopathy Complexes Cooperatively Support Ciliogenesis and Ciliary Signaling
Source: PLoS Genet. 2015 Nov 5;11(11):e1005627. doi: 10.1371/journal.pgen.1005627 (PMC4635004; doi:10.1371/journal.pgen.1005627)
Supplement: S1 Table — The number of limbs with the indicated number of digits observed, with the percentage in parentheses. (PDF) [file pgen.1005627.s005.pdf]

Genotype	Forelimbs (%)			Total forelimbs	Hindlimbs (%)			Total hindlimbs
	5 digits	6 digits	7 digits	examined	5 digits	6 digits	7 digits	examined
**WT**	34 (100)	0 (0)	0 (0)	34	34 (100)	0 (0)	0 (0)	34
Tctn1$^{-/-}$	35 (92)	3 (8)	0 (0)	38	2 (5)	35 (92)	1 (3)	38
Tctn2$^{-/-}$	8 (67)	4 (33)	0 (0)	12	0 (0)	12 (100)	0 (0)	12
Cc2d2a$^{-/-}$	14 (100)	0 (0)	0 (0)	14	2 (14)	12 (86)	0 (0)	24
Nphp4$^{n/n}$	36 (100)	0 (0)	0 (0)	36	36 (100)	0 (0)	0 (0)	36
Nphp1$^{-/-}$	28 (100)	0 (0)	0 (0)	28	28 (100)	0 (0)	0 (0)	28
Tctn1$^{-/-}$ ***Tctn2***$^{-/-}$	17 (94)	1 (6)	0 (0)	18	1 (6)	17 (94)	0 (0)	18
Tctn1$^{-/-}$ ***Nphp4***$^{n/n}$	4 (17)	15 (62)	5 (21)	24	0 (0)	21 (88)	3 (12)	24
Tctn1$^{-/-}$ ***Nphp1***$^{-/-}$	8 (33)	16 (67)	0 (0)	24	0 (0)	24 (100)	0 (0)	24
Cc2d2a$^{-/-}$ ***Nphp4***$^{n/n}$	1 (12)	5 (63)	2 (25)	8	1 (12)	7 (88)	0 (0)	8
