# Supplementary material for: Conserved Genetic Interactions between Ciliopathy Complexes Cooperatively Support Ciliogenesis and Ciliary Signaling
Source: PLoS Genet. 2015 Nov 5;11(11):e1005627. doi: 10.1371/journal.pgen.1005627 (PMC4635004; doi:10.1371/journal.pgen.1005627)
Supplement: S2 Table — The number of embryos with exencephaly among the total examined, with the percentage in parentheses. (PDF) [file pgen.1005627.s006.pdf]

Genotype	Embryos with exencephaly (%)
**WT**	0/17 (0)
Tctn1$^{-/-}$	0/19 (0)
Nphp4$^{n/n}$	0/18 (0)
Nphp1$^{-/-}$	0/14 (0)
Tctn1$^{-/-}$ ***Nphp4***$^{n/n}$	2/12 (17)
Tctn1$^{-/-}$ ***Nphp1***$^{-/-}$	5/12 (42)
