# Supplementary material for: Conserved Genetic Interactions between Ciliopathy Complexes Cooperatively Support Ciliogenesis and Ciliary Signaling
Source: PLoS Genet. 2015 Nov 5;11(11):e1005627. doi: 10.1371/journal.pgen.1005627 (PMC4635004; doi:10.1371/journal.pgen.1005627)
Supplement: S3 Table — The number of limbs with the indicated number of digits observed, with the percentage in parentheses. (PDF) [file pgen.1005627.s007.pdf]

Genotype	Forelimbs (%)			Total forelimbs examined	Hindlimbs (%)			Total hindlimbs examined
	5 digits	6 digits	7 digits		5 digits	6 digits	7 digits	
**WT**	12 (100)	0 (0)	0 (0)	12	12 (100)	0 (0)	0 (0)	12
Tctn1 $^{-/-}$	8 (100)	0 (0)	0 (0)	8	2 (20)	8 (80)	0 (0)	10
Bbs1 $^{-/-}$	12 (100)	0 (0)	0 (0)	12	12 (100)	0 (0)	0 (0)	12
Tctn1 $^{-/-}$ ***Bbs1*** $^{-/-}$	0 (0)	4 (67)	2 (33)	6	0 (0)	6 (100)	0 (0)	6
