# Supplementary material for: Conserved Genetic Interactions between Ciliopathy Complexes Cooperatively Support Ciliogenesis and Ciliary Signaling
Source: PLoS Genet. 2015 Nov 5;11(11):e1005627. doi: 10.1371/journal.pgen.1005627 (PMC4635004; doi:10.1371/journal.pgen.1005627)
Supplement: S4 Table — The number of embryos with exencephaly among the total examined, with the percentage in parentheses. (PDF) [file pgen.1005627.s008.pdf]

Genotype	Embryos with exencephaly (%)
**WT**	0/6 (0)
Tctn1$^{-/-}$	0/5 (0)
Bbs1$^{-/-}$	0/6 (0)
Tctn1$^{-/-}$ ***Bbs1***$^{-/-}$	2/3 (67)
