# Supplementary material for: Conserved Genetic Interactions between Ciliopathy Complexes Cooperatively Support Ciliogenesis and Ciliary Signaling
Source: PLoS Genet. 2015 Nov 5;11(11):e1005627. doi: 10.1371/journal.pgen.1005627 (PMC4635004; doi:10.1371/journal.pgen.1005627)
Supplement: S5 Table — (PDF) [file pgen.1005627.s009.pdf]

Strain	Genotype
**N2**	wild type
**KQ2175**	*tctn-1(ok3021)* IV
**KQ1456**	*nphp-4(tm925)* V
**KQ2206**	*nphp-1(ok500)* II
**KQ2333**	*mks-1 (tm2705)* III
**KQ2209**	*mksr-1(ok2092)* X
**KQ2245**	*mksr-2 (tm2452)* IV
**KQ2185**	*mks-3(tm2547)* II
**KQ2187**	*tctn-1(ok3021)* IV; *nphp-4(tm925)* V
**KQ2232**	*nphp-1(ok500)* II; *tctn-1(ok3021)* IV
**KQ2372**	*mks-1(tm2705)* III; *tctn-1(ok3021)* IV
**KQ2233**	*tctn-1(ok3021)* IV; *mksr-1(ok2092)* X
**KQ2290**	*mksr-2(tm2452)* IV; *tctn-1(ok3021)* IV
**KQ2188**	*mks-3(tm2547)* II; *tctn-1(ok3021)* IV
**KQ1681**	*bbs-5(gk507)* III
**KQ2544**	*bbs-5(gk507)* III; *nphp-4(tm925)* V
**KQ2545**	*bbs-5(gk507)* III; *tctn-1(ok3021)* IV
**KQ2547**	*nphp-1(ok500)* II; *bbs-5(gk507)* III
**KQ2548**	*bbs-5(gk507)* III; *mksr-1(ok2092)* X
**CB1033**	*che-2(e1033)* X
**JT6924**	*daf-19(m86)* II; *daf-12(sa204)* X
**PT709**	*nphp-4(tm925) him-5(e1490)* V
**MX1758**	N2; nxEx[P*bbs-8*::*tctn-1*::gfp + P*osm-5*::*xbx-1*::tdTomato + *rol-6(su1006)*]
**YH372**	N2; Ex[*mksr-1*::cfp + *che-13*::yfp + *rol-6(su1006)*]
**MX1674**	*nphp-4*; nxEx[*mksr-1*::cfp + *che-13*::yfp + *rol-6(su1006)*]
**MX1723**	*tctn-1*; nxEx[*mksr-1*::cfp + *che-13*::yfp + *rol-6(su1006)*]
**MX1726**	*tctn-1; nphp-4*; nxEx[*mksr-1*::cfp + *che-13*::yfp + *rol-6(su1006)*]
**YH751**	N2; yhEx419[P*osm-5*::*mks-6*::gfp + P*osm-5*::*xbx-1*::tdTomato + *rol-6(su1006)*]
**YH776**	*nphp-4*; yhEx419[P*osm-5*::*mks-6*::gfp + P*osm-5*::*xbx-1*::tdTomato + *rol-6(su1006)*]
**MX1675**	*tctn-1*; nxEx[P*osm-5*::*mks-6*::gfp + P*osm-5*::*xbx-1*::tdTomato + *rol-6(su1006)*]
**MX1727**	*tctn-1; nphp-4*; nxEx[P*osm-5*::*mks-6*::gfp + P*osm-5*::*xbx-1*::tdTomato + *rol-6(su1006)*]
**MX1388**	N2; nxEx[*arl-13*::gfp+ P*osm-5*::*xbx-1*::tdTomato + *rol-6(su1006)*]
**MX1751**	*nphp-4*; nxEx[*arl-13*::gfp+ P*osm-5*::*xbx-1*::tdTomato + *rol-6(su1006)*]
**MX1725**	*tctn-1*; nxEx[*arl-13*::gfp+ P*osm-5*::*xbx-1*::tdTomato + *rol-6(su1006)*]
**MX1728**	*tctn-1;nphp-4*; nxEx[*arl-13*::gfp+ P*osm-5*::*xbx-1*::tdTomato + *rol-6(su1006)*]
**YH930**	N2; Ex[P*osm-5*::*mks-5*::tdTomato + P*osm-5*::*dyf-11*::gfp + *rol-6(su1006)*]
**MX1219**	*nphp-4*; nxEx[P*osm-5*::*mks-5*::tdTomato + P*osm-5*::*dyf-11*::gfp + *rol-6(su1006)*]
**MX1722**	*tctn-1*; nxEx[Posm-5::mks-5::tdTomato + Posm-5::*dyf-11*::gfp + *rol-6(su1006)*]
**MX1750**	*tctn-1; nphp-4*; nxEx[P*osm-5*::*mks-5*::tdTomato + P*osm-5*::*dyf-11*::gfp+ *rol-6(su1006)*]
**YH224**	N2; Ex[*nphp-4*::yfp + *che-13*::cfp + *rol-6(su1006)*]
**MX1721**	*tctn-1*; nxEx[*nphp-4*::yfp + *che-13*::cfp + *rol-6(su1006)*]
**MX1420**	N2; nxEx[P*bbs-8*::*tram-1*::tdTomato + P*bbs-8*::*mks-2*::gfp + *rol-6(su1006)*]
**MX1724**	*tctn-1*; nxEx[P*bbs-8*::*tram-1*::tdTomato + P*bbs-8*::*mks-2*::gfp + *rol-6(su1006)*]
**CX3344**	kyIs53[odr-10::GFP] X
**KQ2643**	*nphp-4(tm925)* V; kyIs53[odr-10::GFP] X
**KQ2644**	*tctn-1(ok3021)* IV; kyIs53[odr-10::GFP] X
**KQ2645**	*tctn-1(ok3021)* IV; *nphp-4(tm925)* V; kyIs53[odr-10::GFP] X
